# Loss of residues required for β-arrestin recruitment in the calcium-sensing receptor C-terminal tail may contribute to hypocalcemia

**DOI:** 10.1016/j.jbc.2026.113633

**Published:** 2026-10-09

**Authors:** Rachael A. Wyatt, Hassan Massalha, Julia Drube, Carsten Hoffmann, Caroline M. Gorvin

**Affiliations:** 1 Department of Metabolism and Systems Science, University of Birmingham, Birmingham, United Kingdom; 2 Centre for Membrane Proteins and Receptors (COMPARE), Universities of Birmingham and Nottingham, Birmingham, United Kingdom; 3 Wellcome Sanger Institute, Hinxton, Cambridgeshire, United Kingdom; 4 Theory of Condensed Matter Group, Cavendish Laboratory, University of Cambridge, Cambridge, United Kingdom; 5 Institut für Molekulare Zellbiologie, CMB-Center for Molecular Biomedicine, Universitätsklinikum Jena, Friedrich-Schiller-Universität Jena, Jena, Germany

**Keywords:** calcium homeostasis, endocytosis, hypercalcemia, hypocalcemia, phosphorylation

## Abstract

The calcium-sensing receptor (CaSR) is a family C G protein-coupled receptor (GPCR) with an important role in calcium homeostasis, activating mutations of which cause hypocalcemia. Despite the receptors clinical importance and evidence suggesting β-arrestin-1/2 can modulate CaSR signaling, the residues within CaSR required for β-arrestin recruitment and role of β-arrestin in desensitization, trafficking and signaling are ill defined. We confirmed that β-arrestin-1 and β-arrestin-2 are recruited to CaSR upon receptor activation and that depletion of GPCR kinases impairs β-arrestin recruitment. Deletion of the distal CaSR C-terminal tail, which replicates several mutations identified in individuals with hypocalcemia, reduced β-arrestin-1/2 recruitment and enhanced receptor signaling. Examination of the receptor cytoplasmic region (intracellular loops and C-terminal tail) identified three regions of serine and threonine residues that resemble phosphorylation codes in other GPCRs. Mutation of each of these residues to alanine demonstrated one region between amino acids 1003 to 1011 is important for both β-arrestin-1 and β-arrestin-2 recruitment, while disruption of a region between residues 976 to 981 impaired β-arrestin-2 recruitment. Alanine mutagenesis of these residues also enhanced CaSR signaling and impaired receptor internalization suggesting an important role in receptor desensitization. Three rare-coding variants reported in disorders of calcium homeostasis were identified in these residues and two, T1006M and T1008P, reduced β-arrestin recruitment and CaSR internalization and enhanced CaSR signaling. Thus, we identified two regions within the CaSR C-terminal tail that are important for β-arrestin recruitment, receptor desensitization and internalization. Mutation of residues within these regions may represent another mechanism contributing to hypocalcemia.

The family C GPCR, CaSR, is essential for maintaining calcium homeostasis by regulating parathyroid hormone (PTH) release and urinary calcium excretion (1). Receptor activation elicits signaling predominantly by G_q/11_, stimulating phospholipase C to increase IP_3_-mediated intracellular calcium release (Ca^2+^_i_) and mitogen-activated protein kinase (MAPK) pathways (*e*.*g*., extracellular signal-regulated kinases (ERK1/2)), and G_i/o_ to reduce cAMP 1, 2. For most GPCRs, activation is followed by recruitment of GPCR kinases (GRKs) that phosphorylate the receptor at serine and threonine residues in the intracellular regions (intracellular loops or C terminal tail) to promote interactions with cytosolic β-arrestins (β-arrestin-1 and β-arrestin-2) 3, 4, 5, 6, 7. In the classical model of GPCR signaling, recruitment of β-arrestins physically disrupts G protein coupling and facilitates clathrin-mediated endocytosis of the receptor in a mechanism known as desensitization (3). However, multiple studies have shown that β-arrestins can also act as scaffolds for MAPK signaling 8, 9, 10, and persistent binding of β-arrestin to internalized GPCRs can produce endosomal activation of G protein to sustain receptor responses (11). Although CaSR can produce sustained G_q/11_ signals from endosomes 2, 12, this is by a β-arrestin-independent pathway (12), which has also been demonstrated for other GPCRs (13). Moreover, it is unclear how important β-arrestin recruitment is for CaSR desensitization, signaling and internalization. There is some evidence that β-arrestins are recruited to CaSR and may affect signaling. CaSR coimmunoprecipitates with β-arrestin-1, which was dependent on interaction with the C-terminal tail of CaSR (14). One study showed co-transfection of β-arrestins with GRK2 and GRK4 reduced CaSR-mediated activity (14), while another showed PKC phosphorylation of sites within the C-terminal tail are crucial for β-arrestin recruitment and termination of IP_3_ signaling (15). β-arrestin-1 may also have distinct scaffolding roles in CaSR-mediated cytoskeletal remodeling (16) However, CaSR-mediated phosphorylation of ERK1/2 has been shown to be independent of β-arrestin-2 in one study (17), while another describes its importance for ERK1/2 signaling (18).

Similarly, the importance of β-arrestin recruitment for CaSR internalization has been subject to debate. Studies in HEK293 overexpressing CaSR in the early 2000 s showed agonist-mediated internalization of CaSR was not enhanced by co-transfection of β-arrestin-1, β-arrestin-2, GRK2 or GRK3, nor was there a change in cell surface expression 14, 15, suggesting CaSR trafficking may not require β-arrestin recruitment. Since these studies, there have been substantial developments in assays to assess receptor internalization and a 2019 study that utilized HEK293 depleted of β-arrestin-1 and β-arrestin-2 and a kinetic assay to measure internalization of SNAP-tagged CaSR demonstrated both constitutive and agonist-mediated endocytosis are dependent on β-arrestin-1 and β-arrestin-2 (19). However, they did not investigate the CaSR residues involved in β-arrestin recruitment.

There is significant evidence that different patterns of receptor phosphorylation (the phosphorylation barcode) can induce distinct β-arrestin structural conformations that govern how the receptor signals, is desensitized and internalized 3, 4, 5, 6, 7, 20. Three phosphorylation motifs of different lengths have been described (PxxPxxP/E/D or PxPxxP/E/D or P-x-P-P, where P is a phosphorylated serine or threonine) and proposed to form an extensive network of electrostatic interactions with positively charged pockets in arrestin 6, 20. NMR subsequently demonstrated that GPCR C-termini, which behave as intrinsically disordered regions with transient secondary structures, undergo structural transitions upon GRK phosphorylation to form β-strand conformations in localized segments corresponding to arrestin-interacting regions (7). Typically, family A (rhodopsin family) GPCRs harbor one complete or partial phosphorylation code, while family B (secretin family) GPCRs have at least two phosphorylation codes, consistent with transient interactions between β-arrestins and family A GPCRs, and longer-lived interactions that promote distinct signaling patterns with family B receptors 6, 21. Differential phosphorylation of some GPCRs has also been shown to direct signaling towards distinct pathways (*i*.*e*., they bias signaling) (22). The phosphorylation patterns of family C GPCRs are generally understudied although there is evidence that some members of the metabotropic glutamate receptor (mGluR) family have phosphorylation codes within their cytoplasmic region (23).

The CaSR has a long C-terminal tail of over 200 amino acids (residues 865-1078). Biochemical studies have shown that truncations of the latter part of the C-terminal tail (after residue 886) can enhance receptor surface expression and downstream signaling 24, 25, 26, 27. Consistent with this, several *CASR* truncation mutations that affect this region of the C-terminal tail increase receptor activity and cause autosomal dominant hypocalcemia (ADH) 28, 29, whereas inactivating truncation mutations that cause the opposite disorder, familial hypocalciuric hypercalcemia, cluster in the proximal C-terminal tail, close to the transmembrane region 30, 31. Truncation of the latter part of the C-terminal tail likely enhances signaling by a combination of mechanisms including retention of essential residues for signaling in the proximal C-terminal tail 32, 33 as well as an ER checkpoint motif important for cell surface expression (26), whereas the distal C-terminal tail harbors a dileucine motif required for receptor internalization and motifs that target CaSR to degradation pathways, which when lost will increase signaling 24, 34. However, it is also possible that this distal C-terminal tail also harbors phosphorylation sites that promote β-arrestin recruitment.

Here we investigated which residues in the CaSR intracellular region are important for β-arrestin recruitment. We identified two motifs in the C-terminal tail that may have roles in β-arrestin recruitment and showed clinically relevant variants within these sites can enhance signaling by reducing β-arrestin recruitment.

## Results

### CaSR activation recruits β-arrestin-1 and β-arrestin-2 to the cell surface

We used the NanoBiT β-arrestin assay (35) to determine whether CaSR activation affects β-arrestin-1/2 recruitment in HEK293 cells depleted of β-arrestin-1/2 by CRISPR-Cas gene-editing (HEK-βarr1/2-KO). Cells were transfected with CaSR-SmC, which we have previously shown to express and signal similarly to FLAG-tagged CaSR (2), and LgC-β-arrestin-1 or LgC-β-arrestin-2 and exposed to Ca^2+^_e_ to stimulate the receptor. This showed robust luminescence responses for both β-arrestin-1 and β-arrestin-2 (Fig. 1*A*). Responses were larger for β-arrestin-1, although the EC_50_ values were not significantly different (Fig. 1*B*). Similarly, both β-arrestin-1 and β-arrestin-2 are recruited to CaSR-Nluc measured by bioluminescence resonance energy transfer (BRET) in HEK-βarr1/2-KO and recruitment is greater for β-arrestin-1, although EC_50_ values are not significantly different (Fig. 1, *C* and *D*). Therefore, CaSR can recruit both β-arrestin-1 and β-arrestin-2.

**Figure 1:**
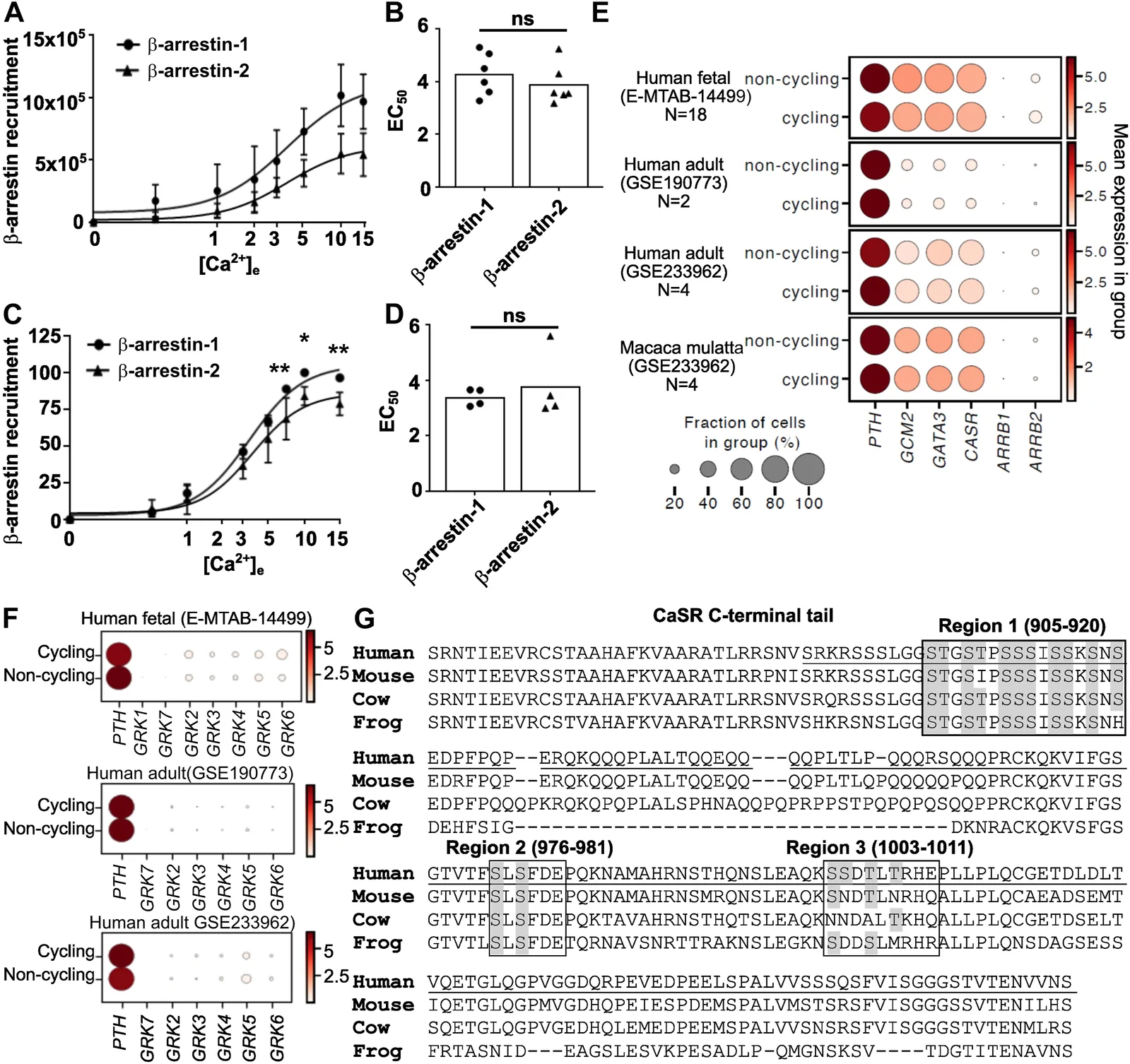
**CaSR recruits both β-arrestin-1 and β-arrestin-2 and there are three predicted phosphorylation regions in the C-terminus.***A*, AUC from luminescence measured in response to increasing concentrations of Ca^2+^_e_ in HEK-βarr1/2 KO cells transfected with CaSR-SmC and either LgC-βarrestin-1 or LgC-βarrestin-2. N = 6 biological replicates. Data shows mean ± SD. *B*, EC_50_ values derived from *panel A*. *C*, normalized BRET between CaSR-Nluc and β-arrestin-1-YFP or β-arrestin-2-YFP measured in HEK-βarr-1/2 KO cells. N = 4 biological replicates. Data shows mean ± SD. *D*, EC_50_ values derived from panel C. *E* and *F*, *dot plot* showing the mean expression of canonical parathyroid cell markers (parathyroid hormone, GCM2, GATA3, CASR) together with (*E*) ARRB1 and ARRB2 and (*F*) GRKs in parathyroid cells from publicly available datasets (accession numbers indicated next to each panel). Expression levels are shown separately for cycling (G2M and S) and non-cycling (G1) cells. *Dot size* indicates the proportion of cells within each category with detectable expression. N denotes the number of individuals included from each study. *G*, multiple sequence alignment of the CaSR C-terminal tail with the three predicted phosphorylation sites shown in boxes and the Ser/Thr residues highlighted in *gray*. Statistical analyses in *B* and *D* were performed by unpaired *t* test and by two-way ANOVA with Sidak’s multiple comparisons test in *A* and *C* ∗*p* < 0.05, ∗∗*p* < 0.01. AUC, area-under-the-curve; BRET, bioluminescence resonance energy transfer; CaSR, calcium-sensing receptor; GATA3, GATA binding factor 3; GCM2, Glial Cells Missing Transcription Factor 2; YFP, yellow fluorescence protein.

β-arrestin-1 and β-arrestin-2 are expressed in human parathyroids (14) although the relative amounts of each gene have not been assessed. We analyzed four single-cell RNA-sequencing datasets containing nonadenoma parathyroid cells from human fetal, human adult and rhesus macaque. Expression of β-arrestin-2 was higher in all samples than β-arrestin-1 (Fig. 1*C*) suggesting CaSR is more likely to couple to β-arrestin-2 in parathyroid cells where it performs its primary function. Notably, cycling fetal parathyroid cells showed higher β-arrestin-2 expression than noncycling cells (approximately 1.4 times higher). Expression of *CASR* was higher in human fetal than human adult cells, with 1.86 to 4.53-fold increase (Fig. 1*E*). GRKs phosphorylate residues on GPCRs that are recognized by β-arrestins and we also analyzed the parathyroid datasets to determine GRK expression. Expression of *GRK1* and *GRK7* were absent, consistent with their retinal-specific roles. *GRK5* had higher expression in fetal and adult parathyroid cells (Fig. 1*F*), although other GRKs were present as described in a previous study (14).

### The distal C-terminal tail of CaSR is required for β-arrestin-1 and β-arrestin-2 recruitment

We used phosphorylation site prediction software to determine possible phosphorylation sites within the cytoplasmic regions (intracellular loops and C-terminal tail) of the CaSR that could be important for β-arrestin recruitment. This identified three regions of serine and threonine residues all within the C-terminal tail (Fig. 1*G*). The first region had seven putative phosphorylation sites, although none adhere to the previously described phosphorylation codes used by many GPCRs 3, 4, 5, 6, 7, 20. The second region between amino acids 976 and 981 (SLSFDE) conforms to a PxPxxP/E/D site (Fig. 1*G*). The third region between amino acids 1003 to 1011 has four putative phosphorylation sites and similarly conforms to the PxPxxP/E/D site (Fig. 1*G*). We first generated a CaSR plasmid with deletion of part of the C-terminal tail that encompasses these three putative β-arrestin phosphorylation sites, retains the Thr888 residue that is required for normal CaSR signaling, and replicates mutations identified in individuals with ADH1 in which there is loss of the C-terminal tail following S895 28, 36 (Fig. 2*A*). The mutant Ser895Stop (895X) CaSR-SmC plasmid was shown to express at cell surfaces and signal similarly to the WT plasmid in both parental HEK293 and HEK-β-arr1/2-KO (Fig. 2, *B* and *C*). CaSR-mediated increases in intracellular calcium (Ca^2+^_i_) were assessed in parental HEK293 cells and HEK-β-arr1/2-KO cells. Responses were significantly greater in parental cells expressing CaSR-895X when compared to cells expressing the WT CaSR plasmid with both Ca^2+^_e_ and spermine as agonist (Fig. 2, *D*–*G*), consistent with previous studies 28, 36. However, no differences were detected in HEK-β-arr1/2-KO cells (Fig. 2, *H* and *I*) suggesting the increase in signaling in parental cells is arrestin-dependent (Fig. 2, *H* and *I*). The recruitment of β-arrestin-1 and β-arrestin-2 to either CaSR-SmC WT or CaSR-SmC 895X was next assessed in NanoBiT assays in HEK-β-arr1/2-KO cells. Loss of the C-terminal tail after S895 reduced recruitment of both β-arrestin-1 and β-arrestin-2 (Fig. 2, *J* and *K*) indicating that this region may be important for receptor phosphorylation and subsequent β-arrestin recruitment.

**Figure 2:**
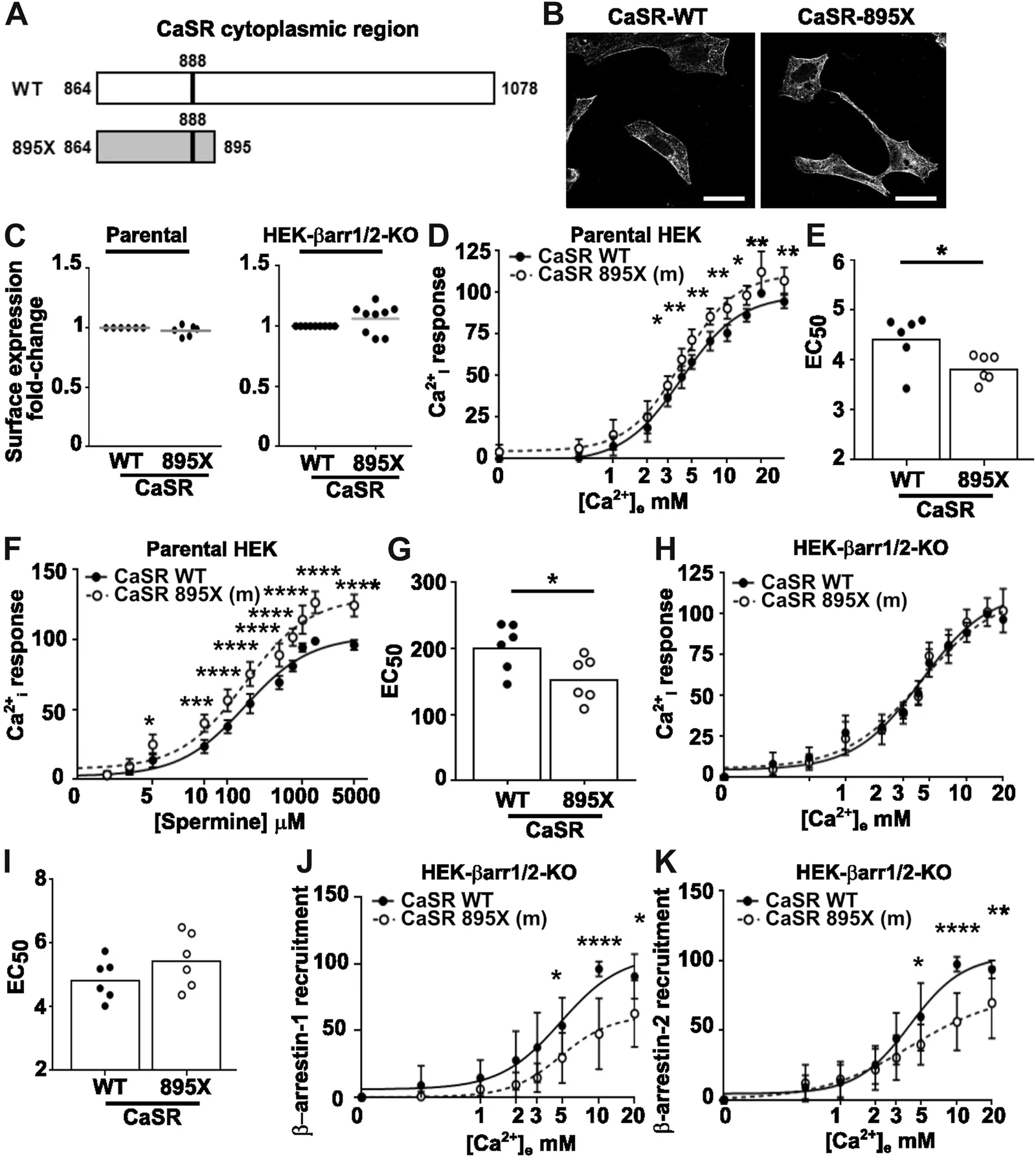
**Loss of the distal C-terminal tail of CaSR enhances signaling and impairs β-arrestin recruitment.***A*, cartoon showing the CaSR C-terminal tail for the WT and 895X truncation plasmid. The Thr888 residue that has previously been shown to have a critical role in CaSR signaling is highlighted and was retained in the truncation plasmid. *B*, confocal images of the CaSR-WT and CaSR-895X plasmids in HEK-βarr1/2 KO cells. *C* and *D*, assessment of cell surface expression measured by labeling surface receptor with a CaSR antibody and a secondary Alexa Fluor 647 antibody in HEK293 and HEK-βarr-1/2 KO cells. Fluorescence was normalized to cells expressing CaSR-WT. *D*, Ca^2+^_i_ responses measured by fluo-4 with (*E*) EC_50_ in response to Ca2+e or *(F* and *G*) with spermine as agonist in cells transfected with CaSR WT or 895X. *H*, Ca2+i responses with (*I*) EC_50_ in HEK-βarr-1/2 KO cells. N = 6 in *D* and *I*. *J*, βarrestin-1 (N = 9) and (*K*) βarrestin-2 (N = 9) recruitment to CaSR in cells expressing CaSR WT and 895X plasmids. AUC was measured to derive concentration-response curves. Data shows mean ± SD in panels *D, F, H, J*, and *K*. Statistical analyses were performed by two-way ANOVA with Sidak’s multiple-comparisons test. ∗*p* < 0.05, ∗∗*p* < 0.01, ∗∗∗*p* < 0.001, ∗∗∗∗*p* < 0.0001. AUC, area-under-the-curve; CaSR, calcium-sensing receptor.

### CaSR has distinct residues for β-arrestin-1 and β-arrestin-2 recruitment

We then investigated the Ser and Thr residues within the three regions predicted to have a role in β-arrestin interactions in HEK-β-arr1/2-KO cells. The seven serine residues within the first β-arrestin phosphorylation site (S911, S912, S913, S915, S916, S918, S920), two serine residues in the second region (S976, S978) and three residues in the third region (S1004, T1006, T1008) were mutated to alanine, NanoBiT recruitment assays performed and compared to WT CaSR responses. Recruitment of β-arrestin-1 was not significantly different to that in cells transfected with WT for all residues in the first and second predicted phosphorylation region (Fig. 3*A* and Table 1). Mutation of all three residues in the third predicted phosphorylation region reduced β-arrestin-1 recruitment at multiple agonist concentrations. EC_50_ values were significantly greater for the S1004A and T1008A variants (Fig. 3*A* and Table 1).

**Figure 3:**
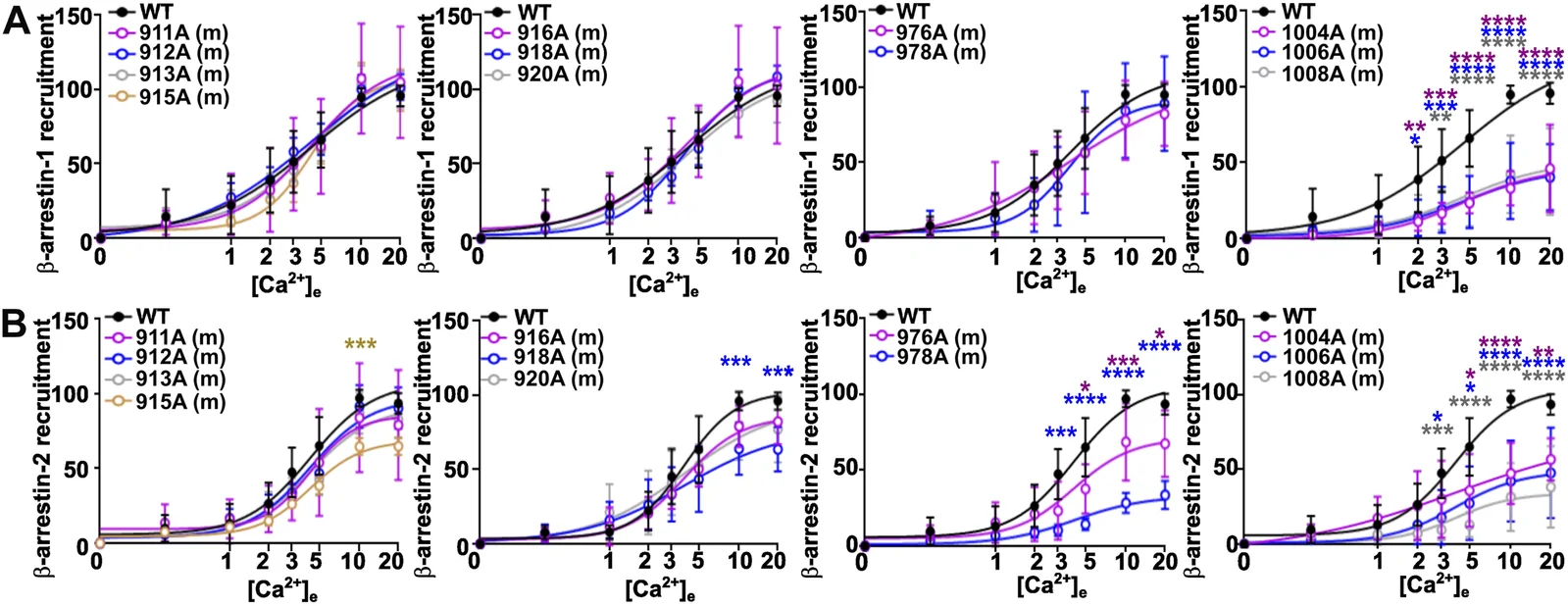
**Mutation of residues in the second and third predicted phosphorylation sites impair β-arrestin recruitment to CaSR.***A*, β-arrestin-1 recruitment and (*B*) β-arrestin-2 recruitment to CaSR in HEK- β-arr1/2-KO cells expressing CaSR WT and predicted phosphorylation site mutant (m) plasmids. AUC was measured to derive concentration-response curves. Statistical analyses were performed by two-way ANOVA with Sidak’s multiple-comparisons test. N = 6 to 8 biological replicates in A and 5 to 8 in B. Data shows mean ± SD. ∗*p* < 0.05, ∗∗*p* < 0.01, ∗∗∗*p* < 0.001, ∗∗∗∗*p* < 0.0001. *Asterisks* in magenta compare WT to 1004A, in *blue* compare WT to 1006A and *gray* compare WT to 1008A. AUC, area-under-the-curve; CaSR, calcium-sensing receptor.

**Table 1:** EC_50_ values for β-arrestin-1 and β-arrestin-2 recruitment and Ca^2+^_i_ assays for WT and mutant plasmids

CaSR	β-arrestin-1 recruitment	β-arrestin-2 recruitment	Ca^2+^_i_ (Ca^2+^_e_ mM)	Ca^2+^_i_ (spermine μM)
WT	3.80 ± 0.28	4.04 ± 0.13	4.26 ± 0.20	216.11 ± 71.87
Ser911Ala	3.94 ± 2.06	4.26 ± 0.26	4.72 ± 0.99	166.77 ± 40.57
Ser912Ala	3.44 ± 0.48	4.92 ± 0.34	4.76 ± 0.75	251.83 ± 100.01
Ser913Ala	4.81 ± 0.56	4.79 ± 0.40	4.87 ± 0.52	208.5 ± 64.10
Ser915Ala	4.57 ± 0.24	4.45 ± 1.77	4.23 ± 0.1.74	268.19 ± 121.34
Ser916Ala	4.39 ± 1.80	4.17 ± 0.13	4.18 ± 0.63	267.74 ± 66.57
Ser918Ala	4.69 ± 1.18	3.42 ± 0.51	4.21 ± 0.56	237.57 ± 99.86
Ser920Ala	3.34 ± 0.66	3.72 ± 0.54	4.64 ± 0.67	201.82 ± 76.28
Ser976Ala	3.81 ± 1.32	4.87 ± 0.31	3.74 ± 0.54^a^	242.25 ± 29.56
Ser978Ala	4.21 ± 0.24	6.65 ± 3.23^a^	3.63 ± 0.64^a^	127.28 ± 65.51^a^
Ser1004Ala	15.91 ± 3.5^a^	6.26 ± 3.58^a^	3.23 ± 0.62^b^	158.6 ± 27.04^a^
Thr1006Ala	4.66 ± 0.93	5.61 ± 2.86^a^	3.80 ± 0.30^b^	126.39 ± 35.24^b^
Thr1008Ala	6.60 ± 3.25^a^	7.10 ± 0.40^a^	3.86 ± 0.30^a^	137.96 ± 72.46^a^

Statistical analyses and pEC_50_ values with SD from concentration-response curves shown in Figures 3 and 4. Analyses were performed by one-way ANOVA with Dunnett’s multiple-comparisons test and compare each group to WT.

a *p* < 0.05.

b *p* < 0.01.

NanoBiT recruitment assays were next performed between β-arrestin-2 and the CaSR mutant plasmids in HEK-β-arr1/2-KO cells. Mutation of six residues (S911, S912, S913, S915, S916, S920) in the first predicted phosphorylation region had little effect on β-arrestin-2 recruitment, although the S918A mutation significantly reduced β-arrestin-2 recruitment without affecting the EC_50_ (Fig. 3*B* and Table 1). Mutation of the two residues of the second region reduced both the Emax and enhanced EC_50_ values for β-arrestin-2 recruitment (Fig. 3*B* and Table 1). Finally, mutation of the three residues in the third predicted region reduced the Emax and EC_50_ values for β-arrestin-2 recruitment (Fig. 3*B* and Table 1). Therefore, mutation of residues in the second and third predicted phosphorylation regions impair both β-arrestin-1 and β-arrestin-2 recruitment, while the second phosphorylation site may also be important for β-arrestin-2 recruitment.

As differences were identified between the two arrestin isoforms, we sought to verify these findings by another method. We generated a CaSR construct with an N-terminal hemagglutinin (HA) epitope followed by a SNAP tag (HA-SNAP-CaSR) that could be used for bystander BRET assays and for subsequent tracking of internalization. Expression of the HA-SNAP-CaSR plasmid at the cell surface was confirmed by structured illumination microscopy (SIM) (Fig. S1*A*). The expression, signaling and trafficking of the plasmid was shown to be similar to expression of the previously described FLAG-CaSR construct (37) (Fig. S1, *B–E*). We then generated alanine mutants of five of the predicted phosphorylation residues in this HA-SNAP-CaSR plasmid, two in site one (915A, 918A), one in site two (978A) and two in site three (1006A, 1008A). Protein expression of the five mutants was similar to the WT HA-SNAP-CaSR plasmid (Fig. S2, *A* and *B*), and all mutants were expressed on the cell surface by HILO (Fig. S2*C*). Bystander BRET assays were performed between Nluc-Kras (to label the plasma membrane) and β-arrestin-1-YFP (yellow fluorescence protein) or β-arrestin-2-YFP in HEK-β-arr1/2-KO cells expressing the CaSR plasmids. Ca^2+^_e_-induced β-arrestin-1 recruitment was impaired by mutation of the T1006 and T1008 residues, while β-arrestin-2 recruitment was impaired by mutation of the S978, T1006 and T1008 residues (Fig. S3). Therefore, both NanoBiT and BRET assays show that β-arrestin isoforms may recognise distinct sites on the CaSR (region 1004-1008 for both β-arrestin-1 and β-arrestin-2, region 976-978 for β-arrestin-2).

### Mutation of CaSR phosphorylation sites affects receptor signaling

As alanine mutagenesis impaired β-arrestin recruitment, we hypothesized that these mutant proteins would affect CaSR signaling. To assess signaling, CaSR-mediated release of intracellular calcium (Ca^2+^_i_) was measured using fluo-4 assays in AdHEK cells expressing either CaSR WT or mutant proteins. Signaling for seven residues was not different to cells expressing CaSR WT plasmids with two CaSR agonists (Ca^2+^_e_ and spermine) (Fig. 4, *A* and *B*). Signaling was enhanced in the two residues of the second predicted phosphorylation site (Fig. 4, *A* and *B*) and the three residues of the third predicted phosphorylation site (Fig. 4, *A* and *B*). Mutation of these five residues significantly reduced EC_50_ values (Table 1), consistent with a gain-of-function. Mutation of these five residues also reduced IP_3_ signaling, measured by IP-1 assays in AdHEK cells (Fig. 4*C*). Therefore, mutation of these C-terminal residues impairs β-arrestin recruitment and enhances CaSR downstream signaling.

**Figure 4:**
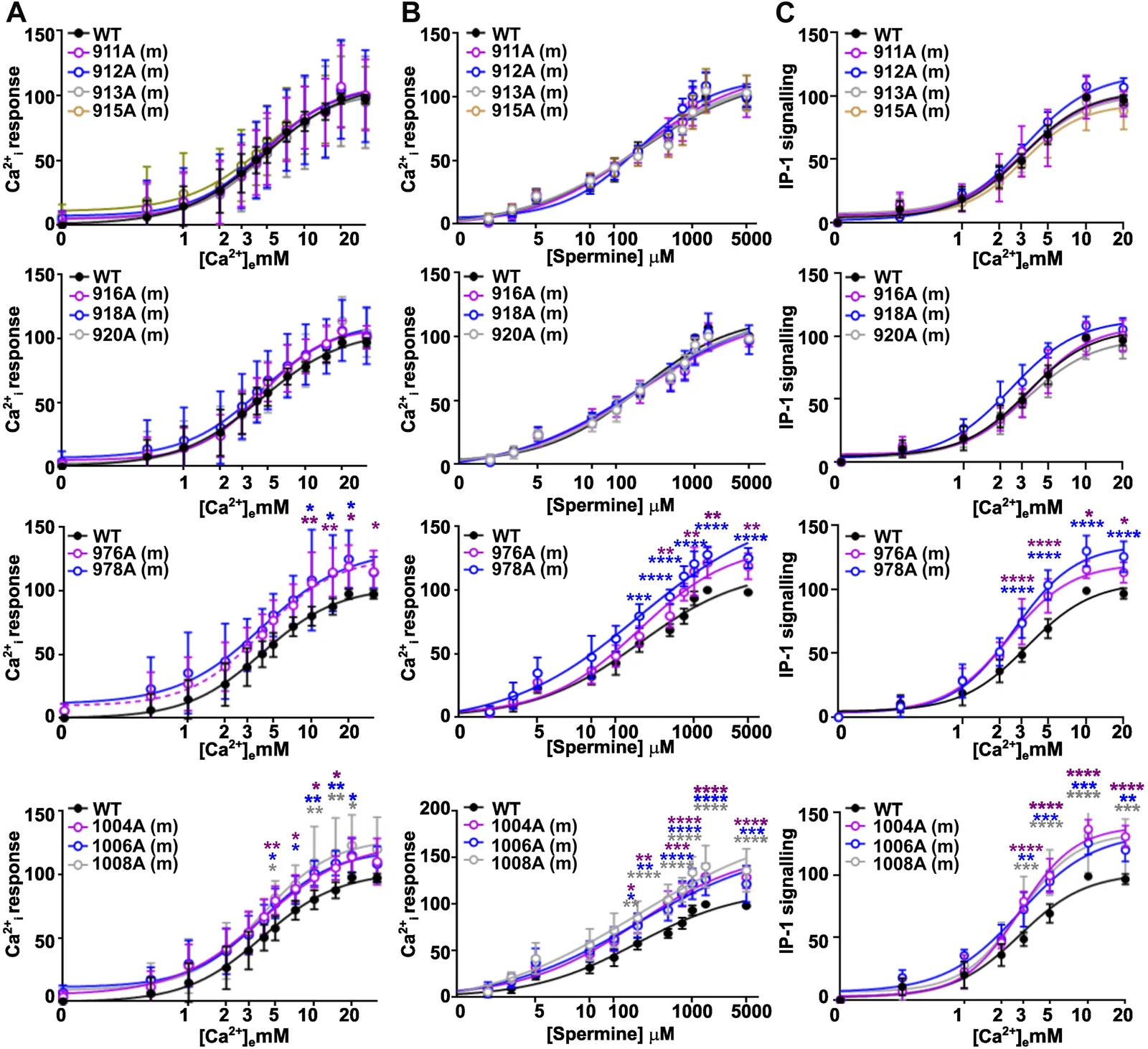
**Mutation of residues in the second and third predicted phosphorylation sites of CaSR enhance Ca^2+^_i_ signaling.** Normalized Ca^2+^_i_ signaling with (*A*) Ca^2+^_e_ and (*B*) spermine as agonist measured by Fluo-4 assays in cells expressing CaSR WT and predicted phosphorylation site mutant (m) plasmids. The maximal response for each drug concentration was derived and normalized to baseline responses of the CaSR-WT transfected wells to derive the concentration-response curve. Statistical analyses were performed by two-way ANOVA with Sidak’s multiple-comparisons test. N = 6 to 9 biological replicates. *C*, CaSR-mediated IP-1 responses in cells expressing CaSR WT and the predicted phosphorylation site mutant plasmids. N = 6 to 7 biological replicates. Data shows mean ± SD. ∗*p* < 0.05, ∗∗*p* < 0.01, ∗∗∗*p* < 0.001, ∗∗∗∗*p* < 0.0001. *Asterisk* color corresponds to comparisons between WT and the mutant labeled with the color in each panel. CaSR, calcium-sensing receptor.

Mutation of these residues may enhance CaSR signaling by preventing β-arrestin recruitment or by other mechanisms. To determine whether these changes in signaling were due to loss of β-arrestin recruitment, IP-1 assays were repeated in HEK-β-arr1/2-KO cells. IP-1 signaling was similar for WT and all CaSR mutant proteins in HEK-β-arr1/2-KO cells (Fig. 5*A* and Table S1), indicating that the enhanced signaling observed in AdHEK cells was likely due to loss of β-arrestin recruitment rather than other mechanisms (*e*.*g*., G protein recruitment).

**Figure 5:**
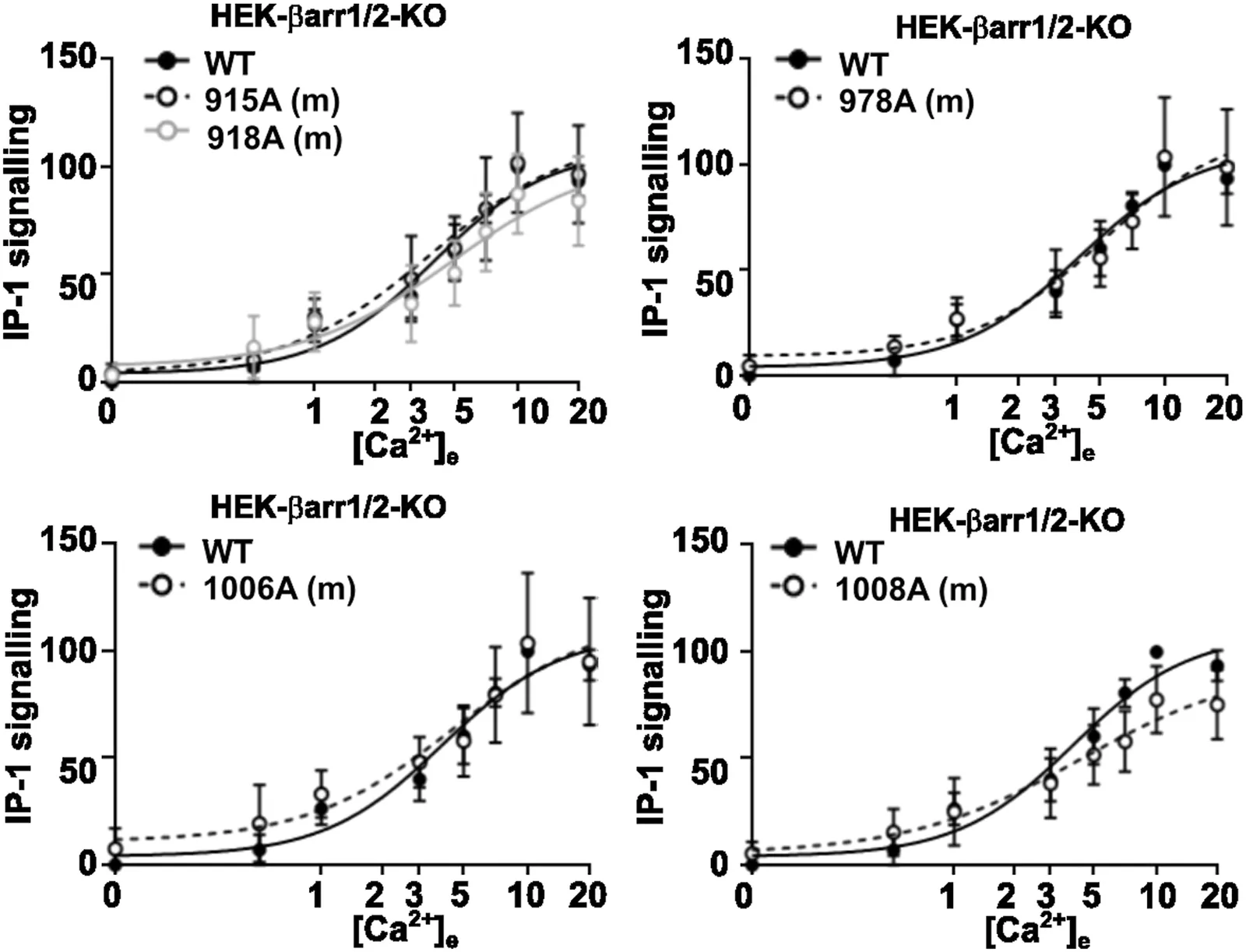
**Enhanced CaSR signaling is lost in cells depleted of β-arrestin-1/2.** CaSR-mediated IP-1 responses in cells expressing CaSR WT and the predicted phosphorylation site mutant (m) plasmids in HEK-β-arr1/2-KO cells. Data shows mean ± SD. Statistical analyses were performed by two-way ANOVA with Sidak’s multiple-comparisons test. N = 6 biological replicates. CaSR, calcium-sensing receptor.

### Mutation of the second and third phosphorylation site affects CaSR internalization

As β-arrestin recruitment was impaired for some residues, we predicted this would reduce CaSR internalization. We used the HA-SNAP-CaSR plasmid as it’s N-terminal HA epitope and SNAP tag allows surface labeling and tracking of internalization. Ca^2+^_e_-mediated internalization of the construct was confirmed by SIM imaging (Fig. S1*E*). To assess CaSR internalization, cells were first exposed to an HA antibody and vehicle or 5 mM Ca^2+^_e_ for 30 min, then labeled with a fluorescent secondary antibody and the number of internalized vesicles quantified in SIM images. There was a significant difference between the number of vesicles under vehicle conditions (*i*.*e*., constitutive internalization) compared to agonist-driven internalization in all groups (Fig. 6, *A* and *B*). However, agonist driven internalization was significantly reduced in cells expressing the 1006A and 1008A mutants compared to cells expressing the CaSR WT plasmid (Fig. 6, *A* and *B*). The basal cell surface expression of CaSR was also significantly greater in cells expressing these two mutant plasmids, as well as the 978A plasmid (Fig. 6*C*). To monitor internalization by a more quantitative method, we labelled AdHEK cells transfected with either WT or mutant HA-SNAP-CaSR with SNAP-647 and measured fluorescence after 30 min incubation with vehicle or agonist. In parental HEK293 cells, consistent with imaging data, the 1006A and 1008A mutants significantly reduced CaSR internalization (Fig. 6*D*). A small, but significant reduction in CaSR internalization in cells expressing the 978A mutant was also detected (Fig. 6*D*). When assays were repeated in HEK-β-arr1/2-KO cells, internalization was impaired in cells expressing WT CaSR compared to parental HEK293 cells (Fig. 6*E*). Moreover, internalization rates were no longer significantly different for the three mutants in HEK-β-arr1/2-KO cells, suggesting the impaired internalization in parental cells was due to reduced β-arrestin recruitment (Fig. 6*E*).

**Figure 6:**
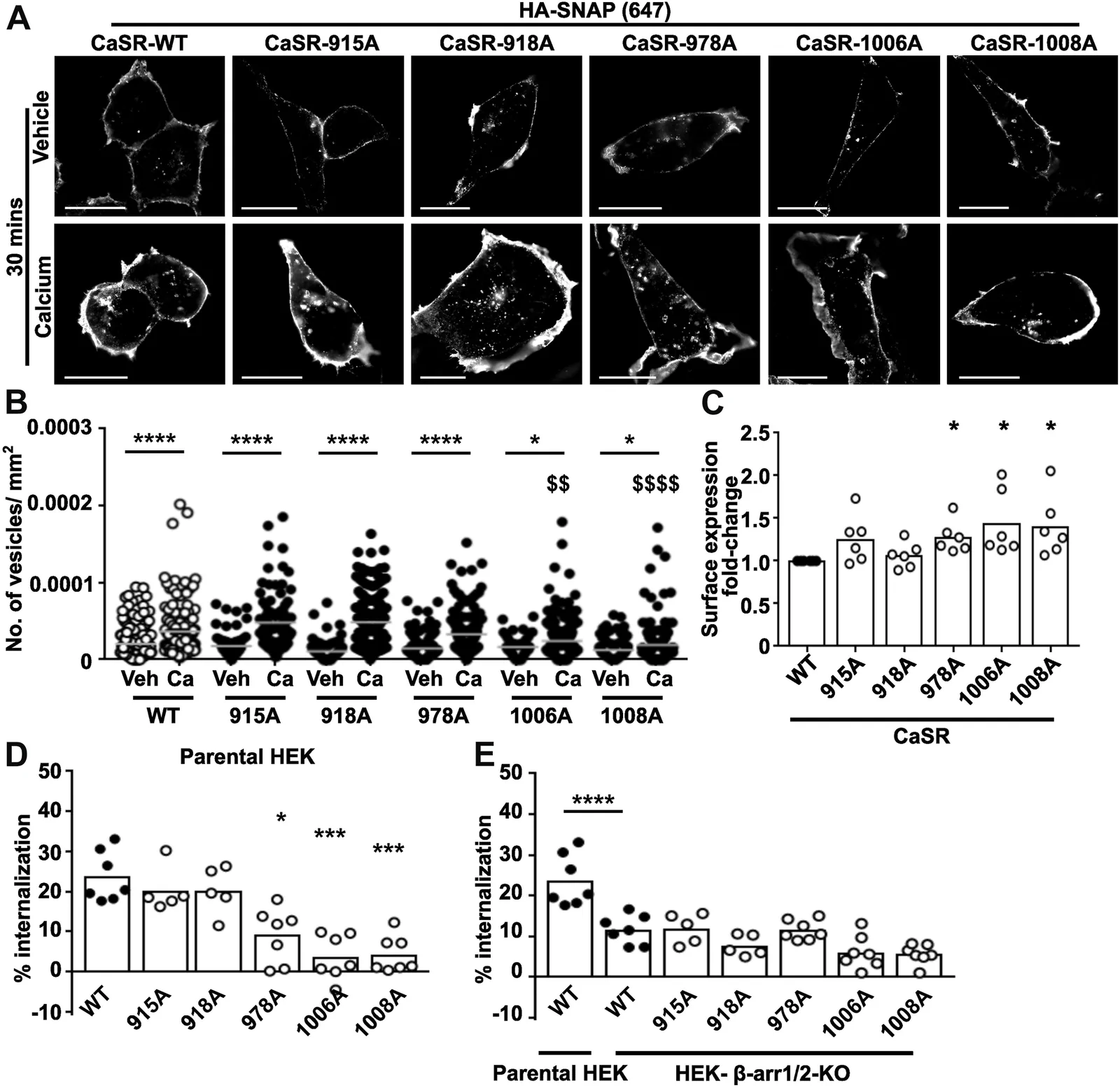
**Mutations in the third phosphorylation site impair CaSR internalization.***A*, SIM of HA-SNAP-CaSR WT and mutant plasmids in HEK293 cells exposed to either vehicle or 5 mM Ca^2+^_e_ for 30 min. The scale bar represents 5 μm. *B*, quantification of the number of internalized vesicles. Number of cells from 4 to 6 independent transfections as follows: WT vehicle (190) and calcium (137), 915A vehicle (70) and calcium (99), 918A vehicle (94) and calcium (159), 978A vehicle (127) and calcium (154), 1006A vehicle (48) and calcium (191), 1008A vehicle (93) and calcium (198). *Gray line* shows mean. *C*, cell surface expression of WT and mutant plasmids. *D* and *E*, percentage internalization of HA-SNAP-CaSR WT and mutant plasmids labeled with SNAP-647 in (*D*) parental HEK293 and (*E*) HEK-β-arr1/2-KO cells. Each dot represents a biological replicate in *C* and *E*. Statistics by one-way ANOVA with Dunnett’s or Sidak’s multiple-comparisons test in *B*, *D* and *E* and compare responses to CaSR-WT in *B and D* and to HEK-β-arr1/2-KO CaSR-WT in E. Statistics by one-sample *t* test compared to hypothetical value of 1 in *panelC*. p-values 0.0145 for 978, 0.0382 for 1006 and 0.0399 for 1008. ∗*p* < 0.05, ∗∗∗*p* < 0.001, ∗∗∗∗*p* < 0.0001. Statistics shown with $ compare agonist responses of each variant with WT calcium responses in *B*. CaSR, calcium-sensing receptor.

### Two regions in CaSR have differential effects on arrestin isoform recruitment and may involve different GRKs

Our β-arrestin recruitment assays suggested different isoforms of β-arrestin may recognise different phosphorylation regions on CaSR. To assess the importance of the second and third phosphorylation regions in further detail, fluorescent internalization assays were repeated in HEK-β-arr1/2-KO cells with overexpression of β-arrestin-1 or β-arrestin-2 to assess the extent to which these rescued CaSR internalization. In HEK-β-arr1/2-KO cells expressing CaSR WT, overexpression of β-arrestin-1 and β-arrestin-2 significantly increased CaSR internalization (Fig. 7*A*). Mutations in the third phosphorylation region impaired recruitment of both β-arrestin-1 and β-arrestin-2 (Fig. 3). Consistent with this region being important for recruitment of both β-arrestin-1 and β-arrestin-2, overexpression of either isoform was unable to enhance internalization in HEK-β-arr1/2-KO cells expressing CaSR 1006A (Fig. 7*B*). Mutations in the second phosphorylation region impaired recruitment of β-arrestin-2 (Fig. 3) and overexpression of β-arrestin-2 was unable to rescue CaSR internalization in HEK-β-arr1/2-KO cells expressing CaSR 978A (Fig. 7*C*). In contrast, β-arrestin-1 overexpression could enhance internalization (Fig. 7*C*) suggesting the S978 residue is not required for recruiting this isoform.

**Figure 7:**
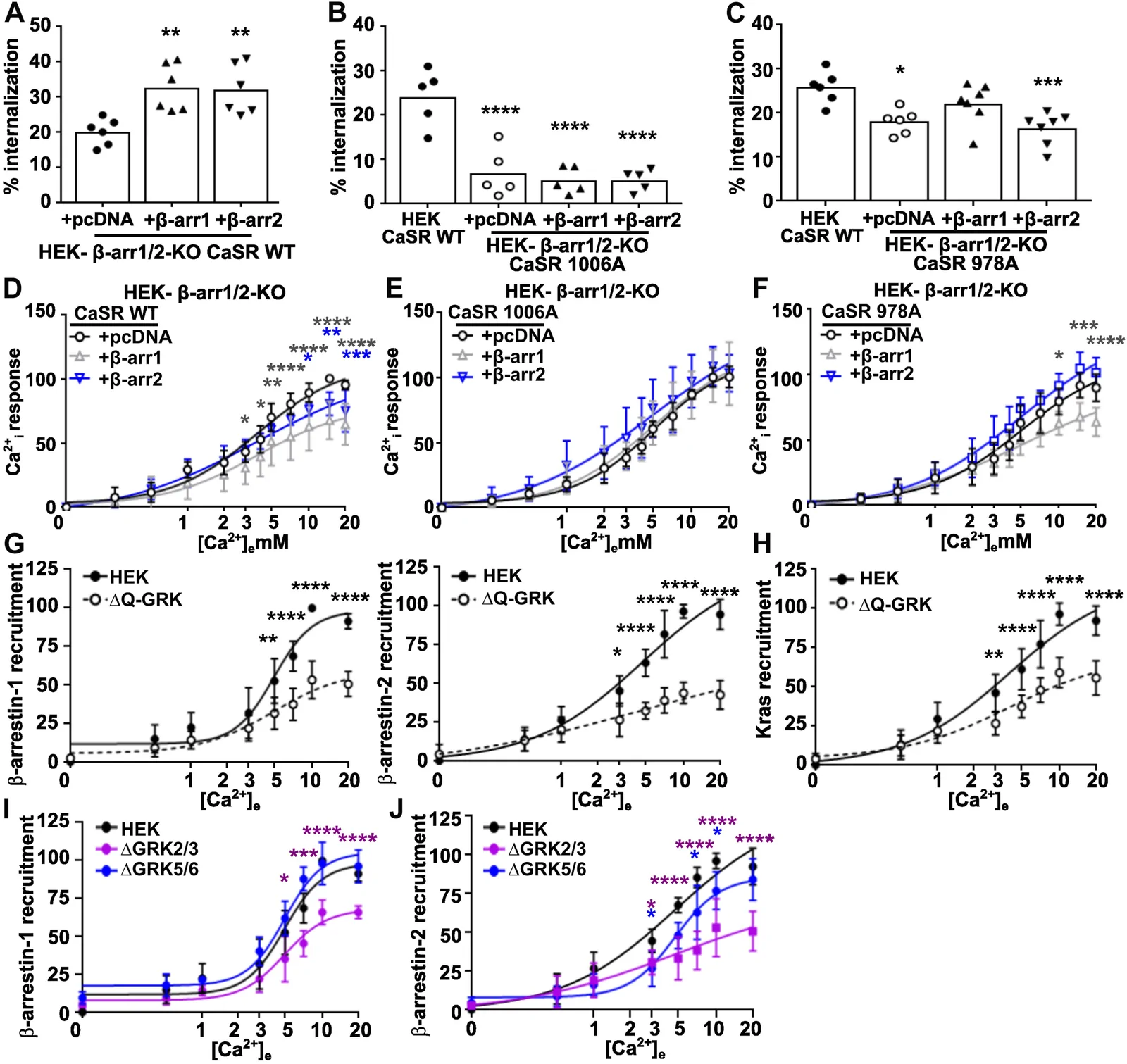
**The second and third phosphorylation regions recruit distinct β-arrestin isoforms and CaSR-mediated β-arrestin recruitment involves GRKs.***A* and *C*, internalization of SNAP-surface-647 labeled HA-SNAP-CaSR (*A*) WT, (*B*) 1006A or (*C*) 978A in cells overexpressing either pcDNA, β-arrestin-1 or β-arrestin-2 over 30 min. Statistical analyses were performed by one-way ANOVA with Dunnett’s multiple comparisons test comparing responses to CaSR WT responses in HEK293. *D and F*, Ca^2+^_i_ signaling in HEK-β-arr1/2-KO cells expressing CaSR (*D*) WT, (*E*) 1006A and (*F*) 978A each with overexpression of pcDNA, β-arrestin-1 or β-arrestin-2. N = 6 to 7 biological replicates. *G*, NanoBiT β-arrestin recruitment assays (N = 6-7 biological replicates) and (*H*) BRET internalization assays (N = 7 biological replicates) in ΔQ-GRK cells or parental HEK293 expressing CaSR WT. *I and J*, CaSR-mediated recruitment of (*I*) β-arrestin-1 and (*J*) β-arrestin-2 to CaSR in ΔGRK2/3, ΔGRK5/6 and parental HEK293. N = 7 biological replicates. Data in *D and J* shows mean ± SD. Statistical analyses in *D* and *J* by two-way ANOVA with Sidak’s multiple-comparisons test. ∗*p* < 0.05, ∗∗*p* < 0.01, ∗∗∗*p* < 0.001, ∗∗∗∗*p* < 0.0001. *Asterisk color* corresponds to comparisons between WT and the mutant labeled with the color in each panel. BRET, bioluminescence resonance energy transfer; CaSR, calcium-sensing receptor.

The ability of each β-arrestin isoform to reduce CaSR signaling was also investigated in HEK-β-arr1/2-KO cells. In cells expressing CaSR WT, CaSR-mediated Ca^2+^_i_ signaling was reduced upon overexpression of β-arrestin-1 and β-arrestin-2 (Fig. 7*D*). In HEK-β-arr1/2-KO cells expressing CaSR 1006A, overexpression of β-arrestin-1 and β-arrestin-2 had no effect on Ca^2+^_i_ signaling responses (Fig. 7*E*), while β-arrestin-1, but not β-arrestin-2 was able to reduce Ca^2+^_i_ signaling in cells expressing CaSR 978A (Fig. 7*F*). Therefore, the third phosphorylation region is likely important for β-arrestin-1 and β-arrestin-2 recruitment, while the second region has a role in β-arrestin-2 recruitment.

Previous studies have shown GRKs are important for arrestin recruitment and GRK isoforms may generate distinct phosphorylation patterns that arrestins recognise differently 3, 38. To assess the importance of GRKs we performed CaSR-mediated β-arrestin recruitment assays in HEK293 cells depleted of different GRK family members (39). Three cell-lines were assessed: ΔGRK2/3, ΔGRK5/6 and ΔQ-GRK2/3/5/6 (ΔQ-GRK), with deletion of the named GRKs, and compared to parental HEK293. CaSR-mediated recruitment of β-arrestin-1 and β-arrestin-2 was impaired in ΔQ-GRK (Fig. 7*G*) and internalization (measured by BRET between Venus-Kras and CaSR-Nluc) was significantly reduced (Fig. 7*H*), indicating that CaSR internalization can be regulated by GRK phosphorylation. Recruitment of β-arrestin-1 was reduced in ΔGRK2/3 cells, but not ΔGRK5/6 cells, while β-arrestin-2 recruitment was reduced in both ΔGRK2/3 and ΔGRK5/6 cells (Fig. 7, *I* and *J*). Therefore, GRK2/3 and GRK5/6 may phosphorylate different regions of the CaSR C-terminal tail, and this may direct recruitment of different arrestin isoforms.

### Rare coding variants impair β-arrestin recruitment and enhance CaSR signaling

Activating mutations in the CaSR are associated with ADH1 and we hypothesized that variants in the CaSR C-terminal tail identified in individuals undergoing investigation for disorders of calcium homeostasis may be associated with changes in β-arrestin recruitment. No ADH1-associated mutations have been described in the literature, therefore we used the ClinVar database to identify germline genetic variants in *CASR* that have been associated with human disease in the residues that are present in the predicted phosphorylation sites. This identified thirteen variants in total affecting eight different amino acids (Table 2). Most variants in the first phosphorylation site were predicted benign and as alanine mutagenesis of these residues did not affect β-arrestin recruitment, Ca^2+^_i_ signaling or CaSR internalization, we did not functionally characterize any variants in these residues. There were no variants in S978 and only a single variant in S978 to a T978 (Table 2). As this variant residue can still be phosphorylated, we chose not to investigate it any further. There were seven variants in the third predicted phosphorylation site (S1004R/C/N, T1006A/R/M, T1008P) and we chose one variant from each residue to characterize in further detail. The CaSR-SmC plasmid was mutated to either 1004C, 1006M or 1008P and β-arrestin recruitment assays performed. The CaSR 1004C variant had no effect on β-arrestin-1 or β-arrestin-2 recruitment (Fig. 8, *A* and *B* and Table 3). In contrast, the 1006M and 1008P variants reduced β-arrestin-1 and β-arrestin-2 recruitment to CaSR, with reduced EC_50_ for 1006M (Fig. 8, *A* and *B* and Table 3). Ca^2+^_i_ recruitment was significantly elevated in cells expressing the 1006M and 1008P variants, with reduced EC_50_ for 1006M, consistent with a gain-of-functional activity (Fig. 8*C* and Table 3). Ca^2+^_i_ signaling in cells expressing the 1004C variant was similar to cells expressing CaSR WT.

**Table 2:** CaSR variants located in the cytoplasmic region identified in the ClinVar database

		Pathogenicity prediction		
CaSR Variant	Source	CADD	SIFT	REVEL
S912F	ClinVar, GnomAD	26.3	predict not tolerated	likely benign
S913F	ClinVar, GnomAD	26.3	predict tolerated	likely benign
S915T	ClinVar	26.1	predict tolerated	likely benign
S920N	ClinVar, GnomAD	18.4	predict tolerated	likely benign
S920R	ClinVar, GnomAD	15.2	predict tolerated	likely benign
S978T	ClinVar	26.3	predict not tolerated	likely benign
S1004R	ClinVar, GnomAD	0.481	predict not tolerated	likely benign
S1004C	ClinVar	22.2	predict not tolerated	likely benign
S1004N	ClinVar	0.094	predict not tolerated	likely benign
T1006A	ClinVar, GnomAD	0.407	predict not tolerated	likely benign
T1006R	ClinVar	16.63	predict not tolerated	likely benign
T1006M	ClinVar, GnomAD	17.9	predict not tolerated	likely benign
T1008P	ClinVar, GnomAD	1	tolerated	likely benign

The ClinVar database was accessed on 28/05/24. All variants identified in the predicted phosphorylation sites are shown and annotated with whether they are also present in GnomAD. Pathogenicity was predicted using three tools, CADD (42), SIFT (43) and REVEL The ClinVar database was accessed on 28/05/24. All variants identified in the predicted phosphorylation sites are shown and whether they are also present in GnomAD listed. Pathogenicity was predicted using three tools, CADD (42), SIFT (43) and REVEL (44). A CADD score greater than 10 indicates the variant is within the 10% most deleterious substitutions, a score greater than 20 indicates the variant is within the 1% most deleterious substitutions.

CADD, combined annotation dependent depletion; REVEL, Rare Exome Variant Ensemble Learner; SIFT, sorting intolerant from intolerant.

**Figure 8:**
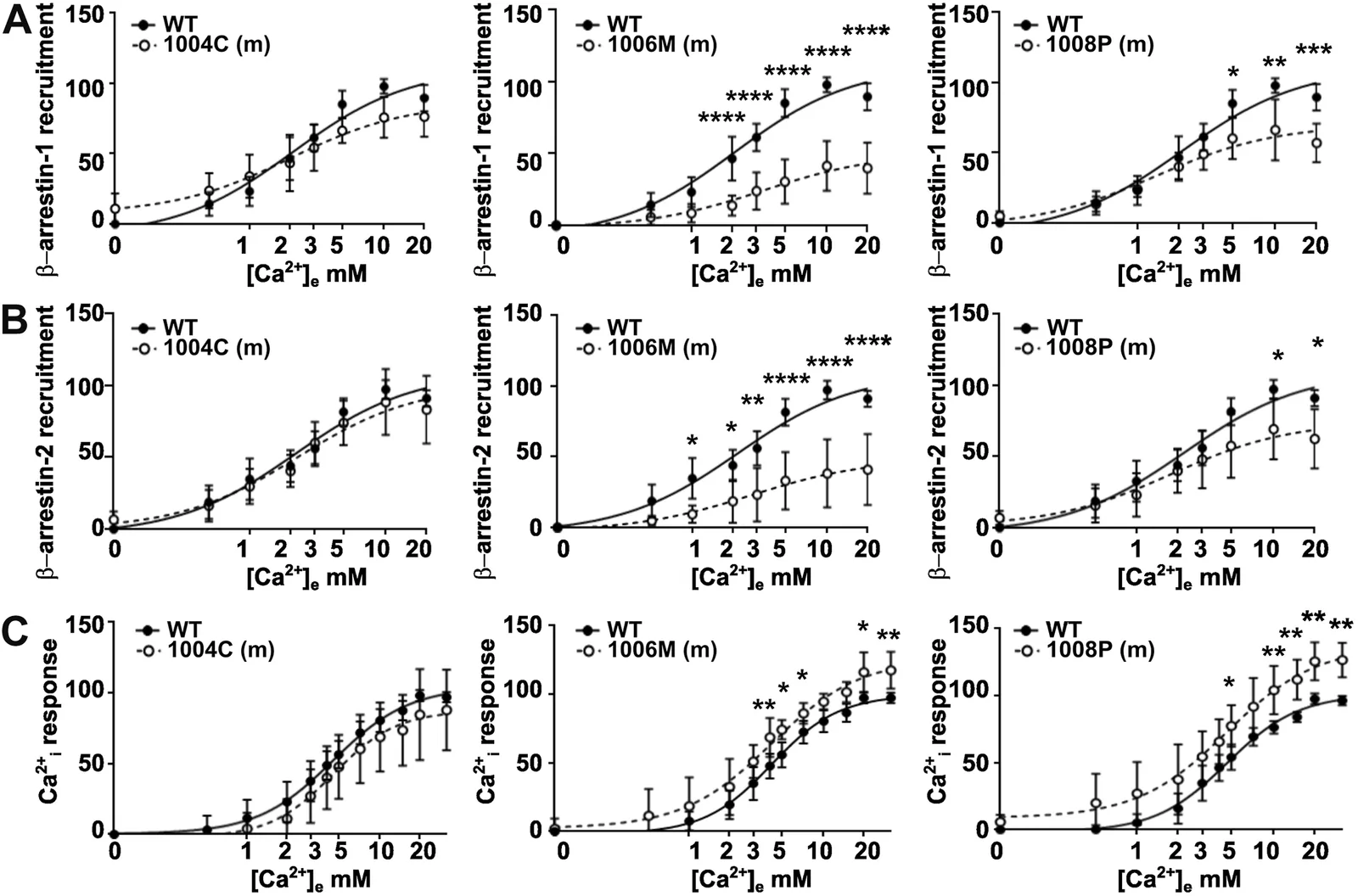
**Rare-coding variants in the third phosphorylation site impair β-arrestin recruitment and enhance CaSR signaling.***A*, β-arrestin-1 recruitment (N = 5) and (*B*) β-arrestin-2 recruitment (N = 7) to CaSR WT and plasmids with the 1006M and 1008P mutants (m) in three residues of the third phosphorylation site. AUC was measured to derive concentration-response curves. *C*, normalized Ca^2+^_i_ signaling measured by Fluo-4 assays in cells expressing CaSR WT and plasmids with ClinVar mutants (m). N = 6 to 7 biological replicates. The maximal response for each drug concentration was derived and normalized to baseline responses of the CaSR-WT transfected wells to derive the concentration-response curve. Data shows mean ± SD. Statistical analyses were performed by two-way ANOVA with Sidak’s multiple-comparisons test. ∗*p* < 0.05, ∗∗*p* < 0.01, ∗∗∗*p* < 0.001, ∗∗∗∗*p* < 0.0001. AUC, area-under-the-curve; CaSR, calcium-sensing receptor.

**Table 3:** EC_50_ values for β-arrestin-1 and β-arrestin-2 recruitment and Ca^2+^_i_ assays for ClinVar mutants

CaSR	β-arrestin-1 recruitment	β-arrestin-2 recruitment	Ca^2+^_i_
WT	2.14 ± 0.69	2.14 ± 0.90	4.33 ± 0.26
Ser1004Cys	2.06 ± 0.88	2.29 ± 0.82	4.58 ± 1.07
Thr1006Met	3.38 ± 0.92^a^	3.33 ± 0.31^a^	3.59 ± 0.12^a^
Thr1008Pro	1.49 ± 0.41	1.87 ± 0.86	4.33 ± 0.67

Statistical analyses and EC_50_ values from concentration-response curves shown in Figure 8. Analyses were performed by one-way ANOVA with Dunnett’s multiple-comparisons test and compare each group to WT.

a *p* < 0.05.

To determine whether these changes in signaling also affected internalization as we observed for the phosphorylation site mutants, we generated a HA-SNAP-CaSR-1006M and a HA-SNAP-1008P plasmid and performed SIM imaging in HEK293 cells. The number of internalized vesicles in cells expressing the 1006M or 1008P plasmid was significantly reduced compared to WT expressing cells following exposure to vehicle or calcium for 30 min (Fig. 9, *A* and *B*), indicating that both agonist-driven and constitutive internalization was impaired in cells with rare-coding variants. To investigate the effects on internalization in further detail and examine β-arrestin-dependent vs. -independent effects we assessed internalization of CaSR labeled with SNAP-surface-647 in HEK293 or HEK-βarr-1/2 KO cells by plate-based assays. CaSR internalization was significantly reduced in HEK293 cells expressing the 1006M and 1008P variants (Fig. 9*C*). Internalization was reduced in HEK-βarr-1/2 KO cells expressing CaSR-WT compared to HEK293 cells (Fig. 9*D*). However, internalization was not further reduced in HEK-βarr-1/2 KO cells expressing 1006M or 1008P compared to HEK293 cells (Fig. 9*C*). There was no significant difference in cell surface expression for the CaSR WT or 1006M and 1008P variants under basal conditions (Fig. 9*D*). As MAPK signaling has been reported downstream of β-arrestin, we also examined the phosphorylation of ERK1/2 (pERK1/2) in response to the 1006M and 1008P mutants and the 1006A and 1008A phosphorylation site mutants. Cells were exposed to Ca^2+^_e_ for 5 and 10 min and pERK1/2 levels assessed by Western blot analysis. When pERK1/2 concentrations were normalized to total ERK1/2 levels, there was no significant difference detected for either the 1006M or 1006A mutants (Figs. 9, *E*–*I*, S4 and S5). Therefore, CaSR rare-coding variants in the C-terminal tail impair β-arrestin recruitment and enhance CaSR Ca^2+^_i_, but do not affect pERK1/2 signaling.

**Figure 9:**
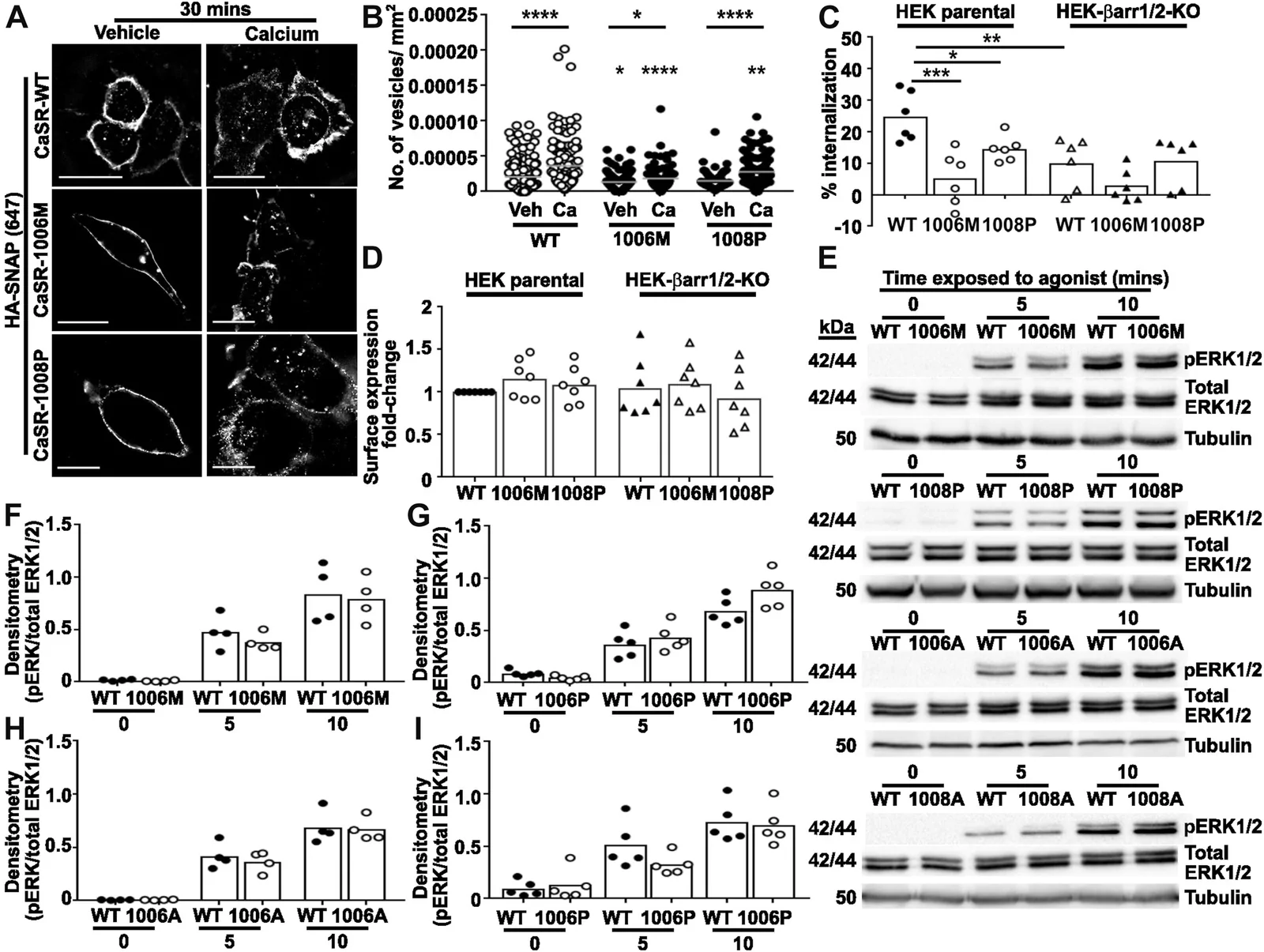
**The CaSR-1006M and CaSR-1008P ClinVar mutants impair internalization but have normal pERK1/2 signaling.***A*, SIM of HA-SNAP-CaSR WT and the 1006M and 1008P mutant plasmids in HEK293 cells exposed to either vehicle or 5 mM Ca^2+^_e_ for 30 min. The scale bar represents 5 μm. *B*, quantification of the number of internalized vesicles. Number of cells from 6 to 7 independent transfections as follows: WT vehicle (190) and calcium (137), 1006M vehicle (119) and calcium (92), 1008P vehicle (66) and calcium (142). *Gray line* shows mean. *C*, assessment of CaSR internalization over 60 min in HEK parental and HEK-β-arr1/2-KO cells expressing the 1006M and 1008P mutants. N = 6. *D*, assessment of cell surface expression of CaSR WT and the 1006M and 1008P mutant. N = 7. Statistics by one-sample *t* test compared to hypothetical value of 1 (*E*) Western blot analysis of phosphorylated ERK1/2 (pERK1/2) in cells expressing HA-SNAP-CaSR WT and 1006M or 1008P mutants and 1006A and 1008A in response to 5 mM Ca^2+^_e_. *E*–*I*, densitometry analysis of pERK1/2 normalized to total ERK1/2 in 4 to 5 blots shown in Figs. S4 and S5. Statistics by one-way ANOVA with Dunnett’s or Sidak’s multiple-comparisons test and compare responses to CaSR-WT. ∗*p* < 0.05, ∗∗*p* < 0.01, ∗∗∗∗*p* < 0.0001. CaSR, calcium-sensing receptor; ERK1, extracellular signal-regulated kinase.

## Discussion

Our studies demonstrated that the CaSR C-terminal tail contains multiple residues that are important for β-arrestin recruitment. Regions in the C-terminal tail were predicted using computational tools, to conform to partial or full phosphorylation sites, and a truncation mutation that resulted in the loss of all three predicted sites impaired β-arrestin recruitment and enhanced receptor signaling. We used alanine mutagenesis of individual residues within these sites to show that β-arrestin recruitment was impaired by introduction of nonphosphorylatable residues within two of these sites. These latter two sites conform to previously described phosphorylation codes of the PxPxxP/E/D format (6). While alanine mutagenesis of three residues in the third site impaired both β-arrestin-1 and β-arrestin-2 recruitment, mutation of S976 and S978 of site two only impaired β-arrestin-2 recruitment. This may be because each arrestin protein recognises different phosphorylation codes and therefore may bind in different conformations to the receptor, as was described for the PTH type-1 receptor (40) and the vasopressin receptor (V_2_R) (41). It is possible that phosphorylation of the two distinct codes allows the CaSR C-terminal tail to adopt different conformations that favor binding of specific β-arrestin isoforms. However, it is difficult to predict how CaSR and β-arrestins interact as the structure of the CaSR C-terminal tail, which is long and intrinsically disordered, has not been determined. Characterisation by structural methods such as NMR may improve our understanding of the CaSR C-terminal tail structure and help predictions of how distinct β-arrestin isoforms interact with CaSR. Our studies showed that different GRKs may phosphorylate distinct regions of the CaSR. Thus, GRK2/3 disrupted β-arrestin-1 and β-arrestin-2 recruitment and suggests GRK2/3 phosphorylates CaSR site 3, while depletion of GRK5/6 disrupted β-arrestin-2 recruitment indicating that it may phosphorylate site two of the CaSR C-terminal tail. Although previous studies have shown GRK-specificity of β-arrestin-1 and β-arrestin-2 to different GPCRs (3), phosphorylation of independent sites by distinct GRKs and recruitment of different β-arrestin isoforms within the same receptor are rare. Such selective recruitment has been shown for the mu-opioid receptor, although this is driven by distinct agonists (42), the PTH receptor-1 (40) and the angiotensin-II type-1A receptor (39), where GRK5/6 targeted residues that β-arrestin-2 is preferentially recruited to. Irrespective of this difference in β-arrestin recruitment, CaSR response to Ca^2+^_i_ was enhanced. Future studies in more physiologically relevant models may help determine whether different GRKs and β-arrestins drive different physiological functions.

We showed that CaSR is capable of recruiting β-arrestin-1 and β-arrestin-2, consistent with previous findings that the receptor interacts with both proteins (14), and that β-arrestin recruitment reduces signaling consistent with the classical model of GPCR desensitization 14, 15, 16. Our analysis of single-cell RNA sequencing datasets showed that β-arrestin-2 expression is higher than β-arrestin-1 and it is therefore likely that CaSR predominantly recruits β-arrestin-2 in physiologically relevant cells. We also identified expression of GRK2-GRK6 in parathyroid cells, consistent with a previous study (14), and we showed greater expression of GRK5, suggesting this may have an important role in parathyroid cells. Our studies noted a higher expression of *CASR* and the β-arrestin genes in human fetal parathyroid than adult parathyroid. Although animal studies suggest these genes are not essential for parathyroid development (43), they may be important for functional maturation during later fetal development. One previous study was unable to identify a role for β-arrestin in CaSR signaling (17); however, they focussed on the ERK1/2 pathway which we similarly showed was not affected by mutation of phosphorylation sites that did impair β-arrestin recruitment. This suggests ERK1/2 signaling from CaSR may be β-arrestin-independent. We have previously shown ERK1/2 can be generated by sustained signaling of CaSR from endosomes 2, 12 and that this pathway is β-arrestin-independent (12). We have verified that the absence of β-arrestin from HEK293 cells does not affect recruitment of Gα_q_ to early endosomes (Fig. S6). Therefore, it is likely that CaSR has both β-arrestin-dependent and β-arrestin-independent pathways that regulate trafficking and signaling, which may explain why depletion of β-arrestin and GRK proteins does not completely abolish CaSR trafficking or signaling in our assays. Collectively, these studies suggest CaSR can perform some signaling in the absence of β-arrestin, similarly to other family C GPCRs, including the GABA_B_ receptor (44) and some mGluRs (23).

We showed that mutation of the second and third predicted phosphorylation motif affected CaSR agonist-driven internalization. While we did not detect a role for the second motif in internalization by one method, a more quantitative method was able to show a reduction in internalization. Moreover, this effect was lost in cells depleted of β-arrestin, suggesting both motifs are involved in β-arrestin recruitment and CaSR internalization to some extent. It is also possible that we detected impairments in internalization more readily for region three mutants as these residues are needed for recruitment of both β-arrestin isoforms, whereas region two is only important for β-arrestin-2 recruitment and β-arrestin-1 may be able to compensate for loss of this site. Although some previous studies of the CaSR suggested that β-arrestin recruitment had no role in CaSR internalization 14, 15, a more recent study that used real-time assays showed β-arrestins did contribute to CaSR internalization (19). It is possible that the earlier studies missed this effect as they used cell surface expression as a readout for internalization 14, 15. Alternatively, it may be because CaSR has considerable constitutive internalization 2, 12, 19, 45 that could have masked agonist-driven effects in these previous studies. Constitutive internalization was not affected by mutation of any of the residues. This contrasts with a previous study that characterized CaSR internalization in HEK293 cells depleted of β-arrestin-1 and β-arrestin-2, which showed both reduced constitutive and agonist-driven internalization (19). However, our assays only looked at the effect of mutation of single residues, not mutation of the whole predicted phosphorylation site, and it is likely that there would be an additive effect of the mutations that would be more analogous to complete depletion of β-arrestin proteins. Previous studies of phosphorylation barcodes have shown such additive effects on the affinity of interactions 5, 7, 41.

Some additional differences were detected between β-arrestin-1 and β-arrestin-2 recruitment to the CaSR. Luciferase values for β-arrestin-1 were higher than β-arrestin-2 in NanoBiT assays and BRET responses were higher, which could suggest β-arrestin-1 forms tighter associations with the receptor. However, there was no difference in the EC_50_ values between the two recruitment assays. It is possible that the differences in NanoBiT assays are due to the position of the LgBiT tag on the β-arrestin proteins, which may not be optimal for recruitment to CaSR, although higher β-arrestin-1 responses were also observed in bystander BRET assays that are less likely to be affected by steric hindrance or other structural artefacts. Other measures of protein affinity would need to be performed to definitively characterize the differences between β-arrestin-1 and β-arrestin-2 recruitment. This is particularly important, as all studies that have assessed receptor affinity have demonstrated β-arrestin-2 has a higher affinity than β-arrestin-1 (46). Mutation of the second and third sites within the C-terminal tail also produced unusual Ca^2+^_i_ signaling curves in which there was an apparent upward shift of the curve, although only responses to high concentrations of Ca^2+^_e_ were significantly different (Fig. 4). Similar responses were seen with spermine as an agonist, although they were not as apparent in IP-1 assays. It is possible that these curves were a consequence of the loss of receptor desensitization, which could produce a slower time course of calcium mobilization. Such effects may not be observed in IP-1 assays as these do not measure immediate signaling effects. It is known that CaSR is more resistant to desensitization than many other GPCRs as it is constantly exposed to its agonist and this may explain why high concentrations of CaSR agonist are required to significantly affect signaling in the β-arrestin mutants.

Deletion of the distal C-terminal tail of the CaSR impaired β-arrestin recruitment and enhanced receptor signaling. Previous studies that also examined truncation of the CaSR C-terminal tail encompassing this region also identified enhanced signaling, attributed to loss of degradation motifs 24, 34. Our studies suggest the enhanced signaling observed in these studies could also be due to impaired β-arrestin recruitment as effects are lost in β-arrestin-null cells. Thus, ADH mutations due to truncations within this region 28, 29 could be associated with receptor activation due to loss of degradation motifs 24, 34, impaired ability to recruit β-arrestin, and loss of an internalization motif. Based on these studies, we hypothesized that other clinically relevant variants in the CaSR may affect β-arrestin recruitment and internalization and that this could modify receptor signaling. However, most disease-associated mutations in the CaSR affect residues within the extracellular domain or transmembrane regions (1), and we instead selected variants from the ClinVar database that have been associated with disorders of calcium homeostasis. A caveat of using this database is that both activating and inactivating mutations in the CaSR are reported as ‘disorders of calcium homeostasis’ and it is not possible to determine whether individuals with these variants are hypo- or hypercalcemic. We assessed three variants affecting residues in the third predicted phosphorylation site and showed that two impaired β-arrestin recruitment and enhanced receptor signaling. Further characterization of these variants demonstrated impaired internalization, consistent with the reduction in β-arrestin recruitment and enhanced CaSR signaling. This suggests that some CaSR variants may affect receptor signaling by modifying receptor phosphorylation and β-arrestin recruitment, similar to disease-causing variants in other GPCRs 47, 48. As CaSR is an obligate dimer it is possible that it may engage multiple β-arrestin proteins simultaneously as was shown for mGluRs (49), and therefore the impact of heterozygote mutations is unknown. Investigation of the calcium levels in individuals with these variants would be useful to determine the relative effects of impaired β-arrestin recruitment, although a large population study may be required to obtain sufficient subjects to determine associations.

There are several limitations in our study. Firstly, experiments were conducted in HEK293 cells with overexpression of the CaSR, which may not reflect the endogenous environment in which CaSR is expressed. CaSR is predominantly expressed in parathyroid chief cells, however, parathyroid organoids are still early in development 50, 51 and only a single rat PTH-C1 has been published (52), but these are not used widely within the research community that study CaSR. Therefore, we used HEK293 cells that are commonly used in the field to examine CaSR and as β-arrestin and GRK KO lines are available for HEK293 35, 39. Another limitation is that we did not specifically show phosphorylation of the CaSR C-terminal sites. Although it is likely the CaSR C-terminal tail is phosphorylated as our assays are sensitive to GRK depletion and previous studies have shown several GRKs regulate CaSR signaling 14, 15, it is possible that alanine substitution could disrupt β-arrestin recruitment through steric or structural mechanisms unrelated to phosphorylation, and direct phosphorylation still remains to be shown for these residues.

In conclusion, our studies have identified two regions within the CaSR C-terminal tail that are important for recruitment of β-arrestin and affect downstream signaling pathways. Clinically-relevant mutations that abolish or disrupt these sites are associated with impaired β-arrestin recruitment and enhanced CaSR signaling and this may represent a novel mechanism by which some ADH mutations cause hypocalcemia.

## Experimental procedures

### Cell culture, transfection and plasmid constructs

Adherent HEK293 (AdHEK) cells were purchased from Agilent Technologies. The authenticity of the cell-line was not independently tested. HEK293 cells with deletion of β-arrestin-1 and β-arrestin-2 (HEK-βarr1/2-KO) were described previously and kindly provided by Asuka Inoue (Tohoku University) (53). HEK293 cells with deletion of different GRK isoforms: ΔGRK2/3 (deletion of GRK2 and GRK3), ΔGRK5/6 (deletion of GRK5 and GRK6) and ΔQ-GRK (deletion of GRK2, GRK3, GRK5, GRK6) have been described previously (39). HEK293 cells and β-arrestin-1/2 KO cells were maintained in DMEM-Glutamax media (Sigma) with 10% fetal bovine serum (Sigma) at 37 °C, 5% CO_2_. Cells were maintained in penicillin-streptomycin-free media and routinely screened to ensure they were mycoplasma-free using the TransDetect Luciferase *Mycoplasma* Detection kit (Generon). Transfections were performed using Lipofectamine 2000 (Invitrogen).

The CaSR-SmC plasmid has been described (2), and the CaSR-SmC-895X truncation was generated by the same method. The NanoBiT expression plasmids, LgC-βarr1, LgC-βarr2 have been described (35) and were provided by Asuka Inoue (Tohoku University). The HA-SNAP-CaSR plasmid was generated within the pRK5-mGluR2 vector (54) (a gift from Joshua Levitz, Weill Cornell Medicine) and has an N-terminal signal peptide followed by the HA affinity tag, SNAP self-labeling protein tag capable of conjugation to organic dyes, and human CaSR (amino acids 20-1078). CaSR-Nluc has been described (2) β-arrestin-1-YFP and β-arrestin-2-YFP were purchased from Addgene (plasmid numbers 36916 and 36,917), Venus-Kras, Venus-Rab5, Nluc-mGq were a gift from Nevin Lambert (Augusta University) (55). SmC-CaSR and HA-SNAP-CaSR mutants were generated using oligonucleotides from Sigma, the QuikChange Lightning site-directed mutagenesis kit (Agilent Technologies) and sequenced by Source Bioscience.

### Bioinformatics resources

Phosphorylation site predictions were made using the Phospho-ELM (56) and NetPhos3.1 (57) resources. Combined annotation dependent depletion (CADD) (58), sorting intolerant from intolerant (SIFT) (59) and Rare Exome Variant Ensemble Learner (REVEL) (60) were used to predict the effect of amino acid substitutions. Amino acid conservation was examined in CaSR mammalian orthologs using ClustalOmega (61). The ClinVar database (62) and the Human Gene Mutation Database (https://www.hgmd.cf.ac.uk/ac/index.php) were examined to identify missense variants in the CaSR C-terminal tail.

### Detection of ARRB1 and ARRB2 in published parathyroid scRNA-seq datasets

Single-cell RNA-sequencing datasets containing parathyroid cells from healthy (nonparathyroid adenoma) human adult 63, 64, human fetal (65), and *Macaca mulatta* (64) samples were included in this analysis. We followed default scanpy integration steps including all cells per study from human adult and *M. mulatta*. For human fetal samples we followed steps as previously described (65). Briefly, basic filtering was applied to post-QC raw count data, retaining cells with more than 500 detected genes and genes expressed in more than three cells. Highly variable genes were then identified using scanpy (v1.11.5, https://scanpy.scverse.org/en/stable/) ‘highly_variable_genes’ (flavor = 'seurat_v3', n_top_genes = 2000). The data for each study were integrated by sample using Harmony (66) (harmonypy; v0.2.0, theta = 0) with the number of principle component analysis dimensions selected based on an elbow plot. For downstream analysis, we subset parathyrocyte clusters based on presence of the canonical marker genes *PTH*, *GCM2* (Glial Cells Missing Transcription Factor 2), *GATA3* (GATA binding factor 3) and *CASR* and assigned a cell-cycle phase to each cell using a cycling gene list from previous studies (67) with the scanpy ‘score_genes_cell_cycle’ function. Normalized expression values are shown per study for noncycling (G1) cells and cycling (S and G2M) cells.

### Assessment of cell surface expression and internalization

For assessment of surface expression of HA-SNAP-CaSR plasmids, AdHEK or HEK-βarr1/2 KO cells were seeded at 10,000 cells/well in black 96-well plates and transfected the same day with 100 ng pcDNA or 100 ng HA-SNAP-CaSR (WT or variants). For overexpression studies, cells were co-transfected with 100 ng of pcDNA, β-arrestin-1-YFP or β-arrestin-2-YFP. Cells were labeled 48-h later with SNAP surface-647, then fluorescence read on a Glomax plate reader (Promega) following three washes with PBS. For internalization, cells were incubated with 5 mM Ca^2+^_e_ or vehicle for 30 min, then fluorescence values read and agonist values normalized to vehicle values. Expression levels were normalized to pcDNA control transfected cells. For assessment of CaSR-895X cell surface expression, AdHEK cells were transfected with 100 ng CaSR-SmC WT or 895X plasmid and cells fixed 48-h later in 4% PFA (Thermo Fisher Scientific) in PBS, then labeled with the mouse monoclonal anti-CaSR primary antibody (1:1000, clone ADD/5C10, Abcam, Cat#ab19347, RRID: AB_444867), followed by Alexa Fluor 647 donkey anti-mouse secondary antibody (Abcam Catalog. number ab181292, RRID:AB_3351687). Cells were washed, then fluorescence read on a Glomax plate reader.

### Western blot analysis

AdHEK cells were seeded into 6-well plates then transfected 24-h later with HA-SNAP-CaSR WT or mutant protein at 1 μg per well. Cells were lysed after 48-h in NP40 buffer and Western blot analysis performed, as described (2). Blots were blocked in 5% marvel/TBS-T, then probed with the primary antibody anti-CaSR (1:1000, clone ADD/5C10, Abcam, Cat#ab19347, RRID: AB_444867) and the Goat Anti-Mouse IgG HRP Conjugated secondary antibody (1:3000, Catalog. no. 1706516, BioRad, RRID:AB_2921252). After visualizing and stripping, the blot was re-probed with the rabbit anti-calnexin polyclonal antibody (1:1000, rabbit polyclonal, Catalog number AB2301, Millipore, RRID:AB_10948000), followed by the Goat Anti-Rabbit IgG, HRP Conjugated secondary antibody (1:3000, Sigma, Catalog no. A0545; RRID:AB_257896).

For pERK1/2 studies, AdHEK cells were transfected with 1 μg plasmid and 36 h later media was replaced with Ca^2+^ and Mg^2+^-free Hank’s Balanced Saline Solution (HBSS). At least 30 min later, cells were stimulated with 5 mM Ca^2+^ for 5 or 10 min, then cells lysed and Western blot performed. After analysis for pERK1/2 (rabbit polyclonal, Catalog no. 9101L, Cell Signaling Technologies, RRID:AB_331646), blots were stripped and re-probed with an anti-total ERK1/2 antibody (rabbit polyclonal, Catalog no. 4695S, Cell Signaling Technologies, RRID:AB_390779), then either rabbit anti-calnexin or rabbit anti-alpha-tubulin monoclonal primary antibody (Abcam, Catalog no. ab176560, RRID:AB_2860019) as loading controls. All antibodies used are well characterized by multiple laboratories. The CaSR antibody has been extensively characterized and shown to be specific for CaSR (68).

Blots were visualized using the Clarity Western ECL kit (BioRad) on a BioRad Chemidoc XRS + system. Densitometry was performed using ImageJ (NIH, https://imagej.net/ij/), and protein quantities normalized to calnexin for CaSR and to total ERK1/2 for pERK1/2 blots.

### NanoBiT β-arrestin recruitment assay

NanoBiT recruitment assays were performed using methods adapted from those previously described 35, 37. NanoBiT β-arrestin assays were performed in HEK-βarr1/2 KO cells or ΔGRK cells. Cells were plated in six-well plates and transfected 48 h later with 500 ng CaSR-SmC WT or mutant plasmids with 200 ng LgC-β-arrestin-1 or LgC-β-arrestin-2.

Following 48 h, cells were harvested in DMEM-FluoroBrite (Gibco) with 10% fetal bovine serum and 4 mM L-glutamine (Gibco) and seeded in eight wells of a 96-well plate. Media was changed to calcium- and magnesium-free HBSS (Sigma) approximately 60 min before the assay was performed. NanoGlo reagent (Promega) was added 15 min prior to assay performance to stabilize baseline reads. Luminescence values were performed on a Promega Glomax at 37 °C. Agonist (CaCl_2_, VWR) was prepared in HBSS at 10x concentration and added to wells once baselines had stabilized, and responses recorded immediately after agonist addition for 35 min. Response values at each concentration were calculated by subtracting the agonist luciferase values from a vehicle-treated sample for each CaSR plasmid, as previously described (69).

Data was plotted using GraphPad Prism 7 (https://www.graphpad.com/features). The area-under-the-curve (AUC) was calculated at each agonist concentration, then the maximal WT response set as 100%, and all mutant responses normalized to this value to compare WT and mutant responses in each experiment. Nonlinear regression of concentration-response curves was performed with GraphPad Prism using the normalized response at each [Ca^2+^]_e_ for the determination of the mean half-maximal concentration (EC_50_).

### BRET assays

NanoBRET assays were performed using methods adapted from previous studies (2). HEK-βarr1/2 KO cells were plated in 96-well white plates and transfected the same day with 50 ng Kras-Nluc, 500 ng Venus-β-arrestin-1 or β-arrestin-2, and 50 ng HA-SNAP-CaSR WT or mutant plasmids. For BRET internalization assays, ΔGRK cells were transfected with 50 ng CaSR-Nluc and 500 ng Venus-Kras. For mGq recruitment studies, HEK293 parental and HEK-β-arr1/2-KO cells were transfected with 100 ng HA-SNAP-CaSR, 500 ng Venus-Rab5 and 50 ng Nluc-mGq. Forty-eight hours later, cells were washed with PBS, then each well was loaded with 40 μl NanoLuc working solution (NanoLuc substrate (Promega) diluted 1:100 in Ca^2+^- and Mg^2+^-free HBSS). BRET measurements were recorded using a GloMax microplate reader (Promega) at donor wavelength 475–30 and acceptor wavelength 535–30 at 37 °C. The BRET ratio (acceptor/donor) was calculated for each time point. Four baseline recordings were made, then agonist added at 8 min and recordings made for a further ∼40 min. The average baseline value was subtracted from the experimental BRET signal. All responses were then normalized to vehicle to obtain a normalized BRET ratio. AUC was calculated in GraphPad Prism and these values used to plot concentration-response curves with a 4-parameter sigmoidal fit.

### IP-1 assays

IP-1 assays were performed using the Cisbio IP-one Gq HTRF kit (Revvity) in AdHEK cells transfected with 100 ng CaSR-SmC WT or mutant plasmids, as described (2). Forty-eight hours later, cells were replated into 384-well plates in Ca^2+^- and Mg^2+^-free HBSS. Agonists were prepared in IP-1 stimulation buffer and incubated for 30 min at 37 °C. Following cell lysis in the supplied buffer, and assays performed according to manufacturer’s instructions. Plates were read on a BMG Labtech PHERAstar measuring emission at 620 and 665 nm and excitation at 320 nm. Data was normalized to baseline levels and concentration-response curves with a 4-parameter sigmoidal fit generated in GraphPad Prism.

### SIM

SIM was performed as previously described (2). HEK293 cells were plated on 24 mm coverslips (VWR) and transfected with 500 ng of each plasmid 36-h prior to experiments. Cells were incubated in Ca^2+^- and Mg^2+^-free HBSS for at least 1 hour, then exposed to 1:1000 anti-HA mouse monoclonal antibody (BioLegend Cat#901514, RRID:AB_2565336) with either vehicle or 5 mM CaCl_2_ for 30 min. Cells were fixed in 4% PFA/PBS, permeabilized with 1% Triton-X100/PBS and exposed to the anti-mouse IgG (H + L), F(ab')2 fragment (Alexa Fluor 647 Conjugate) secondary antibody (1:1000, Cell Signaling Technology, Catalog. no. 4410; RRID:AB_1904023). Samples were imaged on a Nikon N-SIM system (Ti-2 stand, Cairn TwinCam with 2 × Hamamatsu Flash 4 sCMOS cameras, Nikon laser bed 488 and 647 nm excitation lasers, Nikon 100 × 1.49 NA TIRF Apo oil objective). SIM data was reconstructed using NIS-Elements (v. 5.21.03) slice reconstruction. Vesicles were counted in ImageJ using previously described protocols (70). A manual threshold was set for each image to subtract background. Images were processed using ‘Process > Binary > Convert to mask’ and ‘Analyze particles’ in ImageJ. Images were manually inspected to ensure vesicles were captured by this method and observers were blinded to the experimental conditions. The size of each cell was measured in ImageJ and the number of vesicles normalized to the cell area. We have independently validated the HA antibody using cells transfected with a HA-tagged plasmid *versus* nontransfected cells.

### Confocal microscopy

AdHEK cells were plated on coverslips in six-well plates and transiently transfected with 1 μg of either the CaSR-SmC WT or 895X plasmid. Cells were fixed in 4% PFA/PBS, permeabilized with 1% Triton-X100/PBS and exposed to the primary mouse monoclonal anti-CaSR primary antibody (1:1000, clone ADD/5C10, Abcam, Cat#ab19347, RRID: AB_444867), then the secondary antibody Donkey anti-mouse IgG Alexa Fluor-488 (1:1000, Molecular Probes, Cat# A-21202, RRID: AB_141607). Cells were mounted in Prolong Gold Antifade reagent (Invitrogen) and images captured using a Zeiss LSM780 confocal microscope with a Plan-Apochromat x63/1.2/water DIC objective. An argon laser (488 nm) was used to excite Alexa Fluor-488.

#### HILO microscopy

AdHEK cells were seeded on 24 mm coverslips (VWR) and transfected with 500 ng of each plasmid 24-h prior to experiments. On the day of the experiment SNAP-Surface Alexa Fluor 647 (NEB) was diluted 1:1000 in FluoroBrite complete media and applied to cells for 20-min, before washing and imaging. HILO images were acquired on a custom-built TIRF microscope (Cairn Research) containing an Eclipse Ti2 (Nikon) equipped with an EMCCD camera (iXon Ultra, Andor), a 488 nm diode laser, a hardware Perfect Focus System, a TIRF iLas2 module, and a 100x oil-immersion objective (NA 1.49, Nikon). Coverslips were mounted onto plastic imaging chambers with a rubber seal and filled with imaging medium (Ca^2+^- and Mg^2+^-free HBSS with 10 mM Hepes). The objective and samples were maintained at 37 °C. Images were acquired on MetaMorph software (Molecular Devices) using a frame exposure of 50 to 200 ms with two images acquired before ligand stimulation (3 mM Ca^2+^_e_) and a subsequent image taken every 30 s thereafter, up to 20 min. Images were analyzed using ImageJ.

### Fluo-4 intracellular calcium assays

AdHEK or HEK-βarr1/2 KO cells were plated in black sided 96-well plates and transfected with 50 ng CaSR-SmC WT or mutant plasmids per well. For overexpression studies, cells were cotransfected with 50 ng of pcDNA, β-arrestin-1-YFP or β-arrestin-2-YFP. Cells were incubated for 48 h prior to performance of the Fluo-4 intracellular calcium assays. Fluo-4 AM (Molecular Probes, Life Technologies) was resuspended in DMSO/0.03% pluronic acid, then a working solution made on the day of the experiment at 1:1000 dilution in calcium- and magnesium-free HBSS (Sigma) with 10 mM Hepes (Gibco) (HANKS-Hepes solution). Cells were incubated with 50 μl Fluo-4 working solution and incubated at room temperature for 1 hour. Cells were washed once in HANKS-Hepes solution, then 50 μl fresh HANKS-Hepes added prior to assay performance on a Glomax Discover at 37 °C with excitation 494 nm and emission at 516 nm. Ten baseline reads were made per well prior to injection of agonist from preloaded injectors using an automated protocol. Following injection of each agonist concentration, ten reads were performed. At least three technical replicates were performed per condition and the position of each condition varied across each plate. Spermine assays were performed in the presence of 1.5 mM Ca^2+^_e_ (similar to physiological concentrations of calcium). Data was plotted in GraphPad Prism. The maximal response for each drug concentration was derived, normalized to baseline responses of the CaSR-WT transfected wells and concentration-response curves plotted with a 4-parameter sigmoidal fit.

### Statistical analysis

The number of experimental replicates denoted by *n* is indicated in each figure legend. Data were plotted and statistical analyses performed in Graphpad Prism 7. Normality tests (Shapiro–Wilk or D’Agostino–Pearson) were performed on all datasets to determine whether parametric or nonparametric tests were appropriate. A *p*-value of <0.05 was considered statistically significant. A minimum of three independently transfected replicates was performed in all cell-based assays, and each experiment was performed on separate occasions with different passages of cells.

## Data availability

All data needed to evaluate the conclusions in the paper are present in the paper and/or the Supplementary Materials. Raw data will be made available upon request. Plasmid constructs developed for this manuscript will readily be made available upon request. Plasmid constructs obtained from other researchers are detailed in the methods section and may be subject to Material Transfer Agreements. Please contact the corresponding author of this manuscript, or the named source for details.

## Supporting information

This article contains supporting information (58, 59, 60).

## Conflict of interest

The authors declare that they have no conflicts of interest with the contents of this article.
