# Supplementary material for: Loss of residues required for β-arrestin recruitment in the calcium-sensing receptor C-terminal tail may contribute to hypocalcemia
Source: J Biol Chem. 2026 Oct 9;302(11):113633. doi: 10.1016/j.jbc.2026.113633 (PMC13753567; doi:10.1016/j.jbc.2026.113633)
Supplement: Supplementary file 1 [file mmc1.docx]

**Supplementary Appendix**

**Figure S1	Generation of the HA-SNAP-CaSR plasmid**

(**A**) SIM of HA-SNAP-CaSR (labelled with the HA antibody and an Alexa Fluor 647 secondary antibody) and Venus-Kras in AdHEK cells. Scale, 5μm. (**B**) Western blot analyses to detect CaSR in cells transfected with mock (pcDNA3.1), FLAG-CaSR-WT (FLAG) and HA-SNAP-CaSR-WT (CaSR) plasmids showing similar expression levels. Calnexin was used as a loading control. The blot shows lysates from three independent transfections. (**C**) Normalized Ca^2+^_i_ signaling measured by Fluo-4 assays. N=5 biological replicates. The maximal response for each drug concentration was derived and normalized to baseline responses of the CaSR-WT transfected wells to derive the concentration-response curve. (**D**) IP-1 responses in cells transfected with HA-SNAP-CaSR and FLAG-CaSR. N=3 biological replicates. (**E**) SIM images of HA-SNAP-CaSR exposed to 5mM Ca^2+^_e_ and the HA antibody for 0 or 30 minutes. Data shows mean±SD in C and D.

**Figure S2	Total protein expression and surface expression of the HA-SNAP-CaSR WT and mutant plasmids**

(**A**) Western blot analyses to detect CaSR in cells transfected with mock (pcDNA3.1), HA-SNAP-CaSR-WT and mutant plasmids. (**B**) Densitometry of CaSR expression from the five blots normalized to calnexin as a loading control. N=5 biological replicates. Statistical analyses were performed by one-way ANOVA with Dunnett’s multiple-comparisons test. (**C**) HILO imaging of HA-SNAP-CaSR WT and mutant plasmids showing the receptor is expressed at the cell surface. Scale, 5μm.

**Figure S3	BRET assays confirm mutation of the second and third phosphorylation sites differentially disrupt β-arrestin-1 and β-arrestin-2 recruitment to CaSR**

(**A**) β-arrestin-1 recruitment and (**B**) β-arrestin-2 recruitment to CaSR WT or mutant (m) in HEK-β-arr1/2-KO cells measured by bystander BRET. AUC was measured to derive concentration-response curves. Statistical analyses were performed by two-way ANOVA with Sidak’s multiple-comparisons test. N=4 biological replicates in A and 6 in B. Data shows mean±SD. *p<0.05, **p<0.01, ***p<0.001, ****p<0.0001.

**Figure S4	Whole blots for pERK1/2 densitometry shown in Figure 9**

(**A**) Full blots for westerns shown in Figure 9E.

**Figure S5	Whole blots for pERK1/2 densitometry shown in Figure 9**

(**A**) Full blots for westerns shown in Figure 9E.

**Figure S6	Loss of β-arrestin1/2 has no effect on CaSR-mediated recruitment of mGq to Rab5 endosomes**

Bystander BRET measured between Venus-Rab5 and Nluc-mGq in HEK293 parental and HEK-β-arr1/2-KO cells expressing HA-SNAP-CaSR. Data shows mean±SD. N=4 biological replicates.

**Table S1	EC_50_ values for Ca^2+^_i_ assays for WT and mutant plasmids in HEK- β-arr1/2-KO cells**

| **CaSR** | **Ca^2+^_i_**  **(Ca^2+^_e)_** |
| --- | --- |
| WT | 3.97 ± 0.98 |
| Ser915Ala | 3.54 ± 1.78 |
| Ser918Ala | 4.02 ± 1.76 |
| Ser978Ala | 4.35 ± 1.31 |
| Thr1006Ala | 3.90 ± 1.98 |
| Thr1008Ala | 3.86 ± 1.94 |

Statistical analyses and pEC_50_ values with SD from concentration-response curves shown in Figure 5.

**References**

1	Schubach, M., Maass, T., Nazaretyan, L., Roner, S. & Kircher, M. CADD v1.7: using protein language models, regulatory CNNs and other nucleotide-level scores to improve genome-wide variant predictions. *Nucleic Acids Res* **52**, D1143-D1154, doi:10.1093/nar/gkad989 (2024).

2	Sim, N. L. *et al.* SIFT web server: predicting effects of amino acid substitutions on proteins. *Nucleic Acids Res* **40**, W452-457, doi:10.1093/nar/gks539 (2012).

3	Ioannidis, N. M. *et al.* REVEL: An Ensemble Method for Predicting the Pathogenicity of Rare Missense Variants. *Am J Hum Genet* **99**, 877-885, doi:10.1016/j.ajhg.2016.08.016 (2016).
